# What helps or hinders the transformation from a major tertiary center to a major trauma center? Identifying barriers and enablers using the Theoretical Domains Framework

**DOI:** 10.1186/s13049-016-0226-3

**Published:** 2016-03-12

**Authors:** Neil Roberts, Fabiana Lorencatto, Joanna Manson, Susan I Brundage, Jan O Jansen

**Affiliations:** Departments of Critical Care and Anaesthesia, Royal Cornwall Hospital, Truro, Cornwall UK; Division of Health Services Research and Management, School of Health Sciences, City University London, London, UK; Barts Centre for Trauma Sciences, Blizard Institute, Queen Mary University of London, London, UK; Departments of Surgery and Intensive Care Medicine, Aberdeen Royal Infirmary & Health Services Research Unit, University of Aberdeen, Ward 505, Aberdeen, UK

## Abstract

**Background:**

Major Trauma Centers (MTCs), as part of a trauma system, improve survival and functional outcomes from injury. Developing such centers from current teaching hospitals is likely to generate diverse beliefs amongst staff. These may act as barriers or enablers. Prior identification of these may make the service development process more efficient. The importance of applying theory to systematically identify barriers and enablers to changing clinical practice in emergency medicine has been emphasized. This study systematically explored theory-based barriers and enablers towards implementing the transformation of a tertiary hospital into a MTC. Our goal was to demonstrate the use of a replicable method to identify targets that could be addressed to achieve a successful transformation from an organization evolved to provide a particular type of clinical care into a clinical system with different demands, requirements and expectations.

**Methods:**

The Theoretical Domains Framework (TDF) is a tool designed to elicit and analyze beliefs affecting behavior. Semi-structured interviews based around the TDF were conducted in a major tertiary hospital in Scotland due to become a MTC with a purposive sample of major stakeholders including clinicians and nurses from specialties involved in trauma care, clinical managers and administration. Belief statements were identified through qualitative analysis, and assessed for importance according to prevalence, discordance and evidence base.

**Results and discussion:**

1728 utterances were recorded and coded into 91 belief statements. 58 were classified as important barriers/enablers. There were major concerns about resource demands, with optimism conditional on these being met. Distracting priorities abound within the Emergency Department. Better communication is needed. Staff motivation is high and they should be engaged in skills development and developing performance improvement processes.

**Conclusions:**

This study presents a systematic and replicable method of identifying theory-based barriers and enablers towards complex service development. It identifies multiple barriers/enablers that may serve as a basis for developing an implementation intervention to enhance the development of MTCs. This method can be used to address similar challenges in developing specialist centers or implementing clinical practice change in emergency care across both developing and developed countries.

**Electronic supplementary material:**

The online version of this article (doi:10.1186/s13049-016-0226-3) contains supplementary material, which is available to authorized users.

## Background

Effective management of change is central to repositioning of organizations. Even with evidence supporting proposed changes, the development or implementation of new services likely requires active process management to succeed [1, 2]. There are numerous examples of interventions with demonstrated effectiveness that fail to achieve target outcomes when implemented on a large scale, or in new settings [3].

Evidence abounds that high quality care for severely injured patients is best delivered by designated trauma centers within a trauma system. Such service delivery frameworks have been shown to improve survival and functional outcomes [4, 5]. The organizational characteristics of trauma centers typically include strong clinical leadership, integration of services, and organizational commitment [6].

Whilst the United States’ Trauma Systems have evolved significantly over the past 40 years [4, 5, 7], the United Kingdom has lagged behind and this evidence-practice gap has only recently been addressed [8]. London established a Trauma System with four Major Trauma Centers (MTCs, equivalent to “Level 1” trauma centers) in 2010 and various regional systems were launched in England in 2012 [8].

Scotland is currently implementing a national trauma system, with four MTCs to be developed by 2016. Implementing this transformation process in an existing hospital is likely to be highly complex, requiring extensive modifications to infrastructure, processes of care, work patterns and organizational culture.

Successful implementation may be aided by a prior understanding of barriers and enablers to change [9, 10]. Implementation research aims to improve the quality of healthcare and reduce evidence-practice gaps by promoting the uptake of research findings and evidence-based medicine in clinical practice [11]. A recent systematic review of the extent of implementation research in emergency medicine identified that although the number of implementation research papers in this clinical context have significantly increased since 2000, these have primarily focused on identifying evidence-practice gaps [12]. Only a minority of papers reviewed explored barriers and enablers to implementing change in this context. Furthermore, existing studies lacked robust methodologies and designs, with only two studies explicitly using theory [12]. A key recommendation for improving implementation research in emergency medicine thus includes the application of theory to systematically explore barriers and enablers to implementation [12].

The benefits of applying theory to inform the development of interventions has been widely recognized [13], and is advocated by the UK Medical Research Council guidance for developing and evaluating complex interventions [14]. Theory provides a replicable, generalizable framework through which to understand the causal mechanisms underpinning behavior and behavior change [13]. The Theoretical Domains Framework (TDF) is a tool that synthesizes constructs from 33 behavioral theories into 14 theoretical ‘domains’ – such as ‘Knowledge’ or ‘Skills’ – representing a range of possible theory-based barriers and enablers to behavior and behavior change [9]. The TDF aims to simplify psychological theory so that it is accessible to those involved in behavior change research related to the implementation of evidence-based medicine [15]. The TDF can be used as a basis to develop questionnaires or interview topic guides to examine theory-based barriers/enablers. The TDF has primarily been applied to explore barriers and enablers to individual healthcare professional behavior change across a range of clinical contexts, for example, selective gastrointestinal decontamination in critical illness [10, 16]. However, the TDF has not been designed to solely examine individual behavior change, and has more recently also been applied in the context of blood transfusion audit and feedback to investigate barriers/enablers to collective behavior change; that is, multiple healthcare professionals across different organizational levels within a hospital that are collectively involved in the behavior of blood transfusion practice, and responding to transfusion feedback (e.g. consultant hematologists, audit managers, laboratory staff transfusion practitioners, junior doctors) [17]. Barriers/enablers identified through such studies are typically subsequently used as a basis for developing targeted implementation interventions that aim to address the identified barriers/enablers, in order to facilitate the uptake and implementation of evidence-based practice [12, 15].

This study therefore aims to apply the TDF to explore barriers and enablers to implementing the transformation of a tertiary hospital in Scotland into a MTC. Findings will inform strategies to facilitate this implementation within the hospital examined, but also more broadly demonstrate a replicable method for addressing major service redevelopments and the creation of specialist centers and systems.

## Methods

### Design and setting

Semi-structured interviews based on the TDF were conducted to explore barriers and enablers to developing a tertiary hospital into a MTC. The study was conducted in a 900-bed tertiary hospital in Scotland, which already houses the specialties required for MTC status, but has neither trauma surgical services, nor formal systems, guidelines and pathways in place.

### Ethical approval

Approved by Queen Mary University of London Research and Ethics Committee (Reference QMREC1335c) and appropriate local authorities.

### Participants

Transforming a hospital into a MTC is likely to involve multiple specialties and administrative levels. Therefore, to capture a broad spectrum of beliefs from key, relevant, stakeholders, purposive sampling was performed from clinical and management groups. Participants were eligible for inclusion if they currently held a role that would be involved either directly in the care of severely injured patients, or in the transition to a MTC. Such stakeholders groups included: managers, consultants/attendings, trainee doctors, and nurses from relevant disciplines (i.e. emergency medicine, orthopedic surgery, general surgery, anesthesiology, neurosurgery, critical care). There were no additional exclusion/inclusion criteria. Opportunistic sampling techniques were used. Potentially eligible participants were initially identified by an experienced trauma surgeon who worked at the study hospital at the time of data collection. A minimum initial sample size of 10 participants was proposed for full data analysis. An 11th participant was then analyzed, and if new beliefs emerged, sampling continued and data saturation was reassessed in a cyclical manner until achieved. If no new beliefs emerged in the 11th participant their data was discarded. Francis *et al.* in their work on sample size advocate this approach to assessing data saturation in qualitative research [18]. In total, 13 participants were interviewed initially, with a stratified sample of 2 managers, 2 consultants/attendings, 2 trainee doctors, and 2 nurses selected for analysis as part of the first 10 (the minimum sample). The 11th participant analyzed and assessed for data saturation was randomly selected from the remaining three participants.

### Materials

**Fig. 1:**
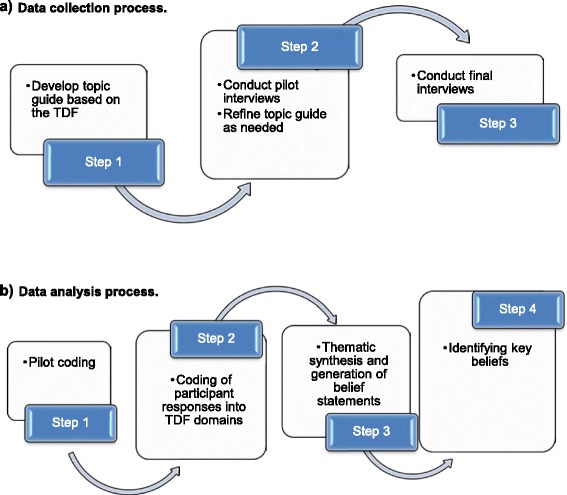
**a** Data collection process. **b** Data analysis process

An interview topic guide containing 43 questions was developed, based on the TDF [9]. The topic guide was structured around three topic areas: 1) participant’s current practice in major trauma care (eg., ‘*How easy or difficult do you find providing major trauma care*?’); 2) participant’s views about MTCs in general (e.g., ‘*What would you see as the benefits of becoming a MTC*?’); and 3) views about the process of transitioning to a MTC (e.g. ‘*Are you aware of any ways in which becoming a MTC is encouraged or rewarded*?’). The topic guide included at least one question related to each of the 14 TDF domains [9]. The TDF provides example questions that may be used to elicit beliefs related to each domain; [9] these were adapted to examine the process of developing a hospital into a MTC. Table 1 lists sample questions from this study for each domain. The topic guide was developed by three clinicians with trauma expertise and a health psychologist. A second health psychologist independently mapped interview questions to TDF domains to validate the original question/domain mapping. Inter-rater reliability was assessed using Fleiss’ Kappa with a minimum value of *k* = 0.75 representing high agreement [19, 20]. Discrepancies were resolved through discussion, and question phrasing revised as necessary. The topic guide was piloted with medical, nursing, and management staff from a district general hospital (A Trauma Unit in the UK system − equivalent to Level III Trauma Center) remote from the tertiary center. This was performed to identify problems such as misunderstanding or repetition, and to optimize question phrasing and order. The topic guide was refined as necessary (Fig. 1a). The resulting topic guide is available online in interview script form as Additional file 1.

**Table 1:** Theoretical Domains Framework (Adapted from Cane et al. 2012)

Domain	Content	Sample question as applied to this study
Knowledge	An awareness of something	In general, how would you describe a major trauma center?
Skills	Ability or proficiency acquired through practice	In general, to what extent do you feel you have the necessary skills or training to contribute to major trauma care?
Social/professional role and identity	Set of behaviors and qualities of an individual in social or work setting	To what extent do you see providing major trauma care as a part of your role?
Beliefs about capabilities	Views about one’s ability/talent/capability to perform the target behavior (s)	How easy or difficult do you find providing major trauma care?
Optimism	Confidence that things will happen for the best or that desired goals will be attained	To what extent do you feel this hospital is ready for the transition to a trauma centre?
Beliefs about consequences	Acceptance of the truth, reality or validity about outcomes of a behavior in a given situation	To what extent do you think the benefits of being a major trauma center outweigh the costs involved in becoming one?
Reinforcement	Increasing the likelihood of a behavior being performed by establishing an association between performing a behavior and a given stimulus or cue	Are you aware of any ways in which becoming a major trauma center is encouraged or rewarded?
Intentions	Conscious decision to perform a behavior or resolve to act in a certain way	Other than the potential transition to major trauma center, are there any changes you are currently planning to make to either you or the hospital’s practice in major trauma care?
Motivation and Goals	Mental representation of outcomes or states that an individual wants to achieve	Are you aware of any goals that have been set for the changing of this hospital into a major trauma center?
Memory, attention and decision processes	The ability to retain information, focus selectively on aspects of the environment and choose between two or more alternatives	Compared with other tasks you have to do in your role, where would you rank contributing towards this hospital’s transition into a major trauma center in terms of priority?
Environmental context and resources	Circumstances of a person’s situation/environment that affect behavior	In general, What resources do you think are required for you in your role to specifically provide major trauma care effectively?
Social influences	Interpersonal processes that can cause individuals to change thoughts/feelings/behaviors	To what extent would you say your general views of major trauma centers are shared by your colleagues in this hospital?
Emotions	Complex reaction pattern by which individual attempts to deal with a personally significant matter or event	Overall, How do you feel about this hospital becoming a major trauma center?
Behavioral regulation	Anything aimed at managing or changing objectively observed or measured actions	How do you and/or your colleagues/team monitor the major trauma care you provide?

### Procedure

Potential participants were identified as key stakeholders by a member of the study team, and formally invited in writing to participate. Interviews were conducted either in person, or by telephone, by a trained interviewer, and recorded digitally. Recordings were transcribed verbatim and anonymized.

### Analysis

Interview transcripts were coded and analyzed in four discrete steps (Fig. 1b), using content analysis following a framework analysis approach [21–24]. These four steps and analysis approaches are standardized methods for analyzing data from interview transcripts based on the TDF, which have been applied in a number of existing semi-structured qualitative interview studies based on the TDF [22–27].

#### Step 1: Pilot coding

To practise applying the TDF and develop coding heuristics to in turn facilitate analysis of transcripts and promote greater consistencies in coding [27], the lead researcher (NR) jointly coded a pilot interview transcript with a health psychologist (FL) using the TDF as a coding framework. Any disagreement or uncertainty was resolved through discussion.

#### Step 2: Coding of participant responses into TDF domains

The lead researcher subsequently coded interview transcripts independently. Using the TDF as a coding framework, participants’ responses within each transcript were split into individual ‘utterances’. These were coded according to which TDF domain they were judged to represent. For example, the statement *“Yes [I’m aware of the developing Scottish trauma system],”* was coded into the domain “Knowledge”. If participant responses concurrently addressed more than one domain, the utterance was allocated to multiple domains (e.g. *“[My colleagues] encourage me to keep [my] technical skills up,”* was coded to both “Social Influences” and “Skills”). A second researcher independently coded a sub-sample of 30 % of transcripts to assess coding reliability to verify whether both researchers would have coded utterances into the same domains. Inter-rater coding reliability was assessed using Cohen’s kappa, with *k* = 0.75 representing high agreement [22]. Discrepancies were resolved through discussion.

#### Step 3: Thematic synthesis and generation of belief statements

In line with a framework analysis approach, Step 3 focused on sifting and sorting of interview data to thematically synthesize and identify key emerging issues [25, 28]. Utterances coded into each domain were compared across transcripts, and utterances expressing similar views regarding potential problems, enablers, or influences on the target behavior were grouped together [27]. A summary belief statement was then generated for each cluster of similar, grouped, utterances. A specific belief statement is defined as ‘a statement that provides detail about the role of the domain in influencing behavior,’ and is intended to represent and summarize shared views that are common across multiple utterances and participants [29, 30]. For example, the utterances *“Outwith the hospital [motivation]’s really variable, actually, outwith the emergency department [motivation]’s really variable.”* and *”…essentially it was because the leadership of surgery didn’t buy into this as a concept. And that’s still the position.”* were grouped and represented by the belief statement ‘Departments and individuals have a high/low motivation for trauma care’. The thematic synthesis was conducted by the lead author (NR) and reviewed by a second author (FL), a health psychologist, to promote robust and defensible coding of data according to theoretical domains [29]. Regular discussions were held to resolve disagreements and reach consensus [22].

#### Step 4: Identifying key beliefs

Each belief statement generated in Step 3 was reviewed against three established criteria [22, 27, 29] to identify key belief statements that are likely to be of greatest relevance/importance for influencing the transition to a MTC. First, total frequency of each belief statement across all interviews; with key belief statements judged to be those expressed by most participants (i.e. highest frequency). Secondly, presence of discordance or conflicting beliefs. Discordant belief statements can help identify specific targets for improvement and develop tailored intervention strategies; for example if some participants knew about goals for transitioning to a MTC, ‘I do (not) know about goals for developing trauma services’ is unlikely to be a barrier for them, but for others who were unaware of these goals, ‘I do (not) know about goals for developing trauma services’ *will* be a barrier for them. Therefore these discordant belief statements tended to represent either barriers for some or enablers for others. Thirdly, the external evidence-base relating to a specific belief statement; if there was an supporting evidence-base relating to an identified belief statement (e.g. belief statement regarding performance improvement processes [6, 7]) it was classified as important. Consensus discussions were held between the research team to review beliefs against these criteria to establish importance, and whether a belief was a barrier, enabler, or conflicting belief which held potential to be both. If a belief statement met any one criterion it was classed as important, if it met none it was classed as unimportant.

## Results

### Participant characteristics

No new belief statements were identified after analysis of the initial 10 participant interviews; therefore, thematic data saturation was deemed achieved and no further interviews were conducted or analyzed (Data saturation table in Additional file 2). The majority of participants were male (60 %). Four participants were consultants, two trainee doctors, two nurses, and two managers, from Intensive Care, Anesthesia, General Surgery, Orthopedics, Neurosurgery and the Emergency Department, and representing management and planning. Participants had a mean of 13 years experience of working at the study hospital (range 8 months-26 years). Interviews with the sample were all performed face-to-face, over a 3-day period, and lasted a mean 53 min (range 28–71 min).

### Coding of participant responses into TDF domains (Analysis Step 2)

Fleiss’s *kappa* for inter-rater reliability in domain-question mapping for the topic guide was 0.75, representing high agreement [19, 20].

In total, 1728 participant responses/utterances were coded into TDF domains. Cohen’s *kappa* for inter-rater reliability averaged across three randomly selected transcripts was 0.67 (range 0.58-0.72) representing substantial agreement as to which domain (s) these utterances represented [31].

Extracted utterances were synthesized into 91 belief statements *(Analysis Step 3),* of which 58 were classified as important barriers and/or enablers *(Analysis Step 4)*. Key belief statements that have been classified as barriers, enablers, or both will be discussed in turn.

### Key barriers

Fourteen belief statements were identified as barriers (Table 2).

**Table 2:** Summary of belief statements classified as barriers

Belief statement (*n* = number of participants expressing the belief)	*n*	Example quote
Knowledge		
Others have variable or limited knowledge of trauma	5	but the level of knowledge that people have of major trauma, of a major trauma center, and the implications of being a major trauma center, uh, are, are, are limited just now, but that’s, that’s what, something we’d need to work on (Manager)
I do not know what the resource requirements are for current trauma care or for becoming a MTC	4	I’m quite sure we don’t have the, the resources in terms of staffing and infrastructure, um, but I don’t know what those are yet, because we haven’t calculated that, but we’re just in the process of doing that. (Manager)
Skills		
In general, there are not sufficient levels of the necessary technical skills at the hospital to provide major trauma care	10	I mean certainly I think experience is lacking, it is just one, uh, is one aspect, um, I think certain courses I think would be useful to do, but I think the fact that, um, I don’t see a huge number of trauma cases but I think my experience is much less of that than someone who is based at a trauma center at the moment. (Registrar)
There are not sufficient amounts of teaching and training in trauma care at the hospital	8	it would be much nicer if we, if we prioritized and nurses did have proper training. Things like the trauma, rather than how to clean a bed frame properly. (Nurse)
Maintaining skills is important as well as developing them	4	Um, trauma for those, because it doesn’t happen, a number of times every day, there is an issue of maintaining skill and making sure that folk are adequately prepared to be able to mount the right response when it is required. (Manager)
Social/Professional Role and Identity		
*No belief statements*		
Beliefs about Capabilities		
Sometimes I require others to help me perform parts of my role in looking after major trauma patients	6	…one individual I think will never have either the skills or the ability to multi-task sufficiently to deal with all aspects of it, so, um, I can deal with, um, a given role, but the big thing is getting people with different skill sets involved… (Consultant)
Optimism		
*No belief statements*		
Beliefs about Consequences		
*No belief statements*		
Reinforcement		
*No belief statements*		
Intentions		
*No belief statements*		
Motivation and Goals		
*No belief statements*		
Memory, Attention and Decision Processes		
There are numerous potential distracting priorities at the same time as trauma that do not allow me to do my job and impact on patient care	9	we’re so busy elsewhere dealing with cases that shouldn’t be coming through the emergency department in order to keep the department safe (Registrar)
Environmental Context and Resources		
We (do not) currently have enough levels of resources to provide good trauma care	10	A lot of our patients, the physio and OT service, as I said, it’s priority of who’s getting, you know, seen, rather than everybody who should be seen is seen. (Nurse)
Substantially more staffing and resources, and maintenance of those already in place, would be required to effectively become a MTC	10	We would need to retain the speciality surgical services, such as cardiothoracic, such as neurosurgery, such as vascular… (Consultant)
The hospital is not organized in the optimum manner for trauma care and a reorganization would improve this	10	…ways of looking at how many people need to be on a trauma rota, so I don’t expect every general surgeon to want to do trauma, um, but if they are happy to facilitate a reasonable number to be on a rota to give that kind of level of response… (Consultant)
It’s not clear how much becoming a MTC will cost or benefit, and funding it may be difficult	8	If there are finite resources, and infinite demands, then somebody will have to make some compromises somewhere. And that’s what the managers and the financiers will have to look into. (Consultant)
Social Influences		
There is variation amongst the views of myself and my colleagues about the transition to a MTC	9	[how committed are your colleagues to becoming a MTC?] The same. But they share my reservations, so, you know, there’s heaps of reservations along the way, but absolutely committed. Just wish we saw that level of commitment from, from everyone. (Consultant)
Management, nursing and medical staff do not work well together at present	3	a lot of the issues that surround us at the front lines, seem to be belittled or ignored by senior management. (Consultant)
Emotions		
*No belief statements*		
Behavioral Regulation		
I do not attend local governance meetings	2	…I used to attend when I could, our M and M meeting you know, with the four consultants, but at the minute there’s like sixteen-odd consultants and growing more and more by the day, so, it’s difficult to attend and be part of that group and understand the, what’s coming back from that morbidity, you know, um, conversations that they have… (Nurse)

#### Environmental context and resources (Four belief statements)

Highly frequent belief statements in this domain related to having insufficient resources to provide major trauma care, and a need for more resources in order to become a MTC. Participants were not sure how much becoming a MTC would cost or how it would be funded, and believed that parts of the hospital were currently poorly organized for major trauma care.

#### Skills (Three belief statements)

Highly frequent belief statements included not only insufficient technical skills to provide major trauma care, but insufficient amounts of teaching and training in trauma care. A less frequent belief described the need for and difficulty in maintaining these skills after developing them.

#### Social Influences (Two belief statements)

Participants described a highly frequent barrier of variable views amongst their colleagues around the transition to MTC. A less frequent belief described a poor working relationship between management, nursing and medical staff at present.

#### Knowledge (Two belief statements)

Participants described moderately frequent barriers of a variable knowledge of trauma care itself, and poor knowledge of the resource requirements for providing trauma care or transitioning to a MTC.

#### Behavioral regulation (One belief statement)

A low frequency belief expressed by nursing staff described a lack of attendance at performance improvement and governance meetings.

#### Belief about capabilities (One belief statement)

A moderately frequent belief statement described the perceived requirement for help from others when a participant cares for a major trauma patient.

#### Memory, attention and decisions (One belief statement)

A highly frequent belief statement described numerous potential distractions and alternate priorities affecting the participant caring for trauma patients.

#### Social/Professional Role and Identity, Optimism, Beliefs about Consequences, Reinforcement, Intentions, Motivation and Goals, Emotions

No belief statements classified as important barriers were mapped to these domains.

### Key Enablers

Twenty belief statements were identified as enablers (Table 3).

**Table 3:** Summary of belief statements classified as enablers

Belief statement (*n* = number of participants expressing the belief)	*n*	Example quote
Knowledge		
I keep up to date with evidence for major trauma care	9	…certainly more so recently. Uh, because of the, the development. (Manager)
I know about trauma care and how to manage trauma patients	5	I think the basics of the, the skills and knowledge, um, that are required for the management of major, major trauma patients, are very much established in, in what we do in critical care. (Consultant)
Skills		
There are sufficient levels of the necessary non-technical skills at the hospital to provide major trauma care	8	I think I’m a good communicator, I get on really well with the staff here. (Registrar)
We can improve our care by learning skills from others both within and outwith trauma	7	Some surgical procedures and, I think that the, it would be of benefit for us to see how it is done elsewhere. (Consultant)
Skills in major trauma care would be better if the hospital were to become an established MTC	7	I think that for trainees, I think it would be hugely useful, I think the experience they would gain from it, I think the decision-making skills, I think the technical skills, um, I think um, I think that that would be fantastic… (Registrar)
Managing trauma patients is routine	7	Yeah. Yeah. I mean, well obviously all the staff are trained up on spinal injuries and, and major injuries like that. They are routine. (Nurse)
Social/Professional Role and Identity		
I should play a role in the transition to major trauma center	8	I now see it as quite a large part of my role, the, uh, because the development of [this hospital] as a major trauma center is regarded as a high priority by the board. (Manager)
I should play a role in the initial assessment and resuscitation of the patient	6	I think [surgeons] should be involved from the outset when they arrive in hospital, um, and I think they should be involved in the decision making for that patient. (Registrar)
Someone should lead and coordinate the care of trauma patients through hospital	5	I think that that would ideally work best because the ownership for the care of that patient would be coordinated by one member or one team, um, which would make it I think much easier to manage them (Registrar)
Beliefs about Capabilities		
We are capable of improving our practice and changing our culture to become a MTC, though it may be difficult in places	9	Well I think the institution of an appropriate group trauma call system. I don’t think that would take very long to, to, um, plan and implement, decide who you need, and then simply get a standard call system for that (Consultant)
Optimism		
*No belief statements*		
Beliefs about Consequences		
Becoming a trauma center would lead to better patient care (more resources, higher priority, more patients, better recruitment)	10	I think the benefits are that we, that we can, uh, build an infrastructure and an image around it which becomes attractive, um, to, to recruiting the best staff we can. So there’s a good reason to come here, because we’re a major trauma center. (Manager)
A co-ordinated approach to efficiently meeting and treating trauma patients would make outcomes better	4	I think that would be really useful because a lot of time is spent looking to see what bleep number is this, this and this, and that's very time-consuming. (Registrar)
Becoming a trauma center would improve staff morale	3	it will boost the morale of the staff employed here. They feel that they are doing something important, they feel valued. They will be able to work as a team, which will be further boost to their morale. (Consultant)
Reinforcement		
*No belief statements*		
Intentions		
*No belief statements*		
Motivation and Goals		
I am motivated to be involved in the transition to MTC	10	I very much feel that we should become a major trauma center, I feel I am committed to doing whatever I could do to facilitate that process, and I would hope that that view is shared by other people. (Consultant)
We should aim to deliver our best care and improve on it	6	I think providing a great service to our patients, I think, um, is something that we should all strive for. (Registrar)
Goals related to trauma care should be a high priority	6	Major trauma care takes priority. First and foremost. (Registrar)
Memory, Attention and Decision Processes		
*No belief statements*		
Environmental Context and Resources		
*No belief statements*		
Social Influences		
Good teamwork is important to the current and future care of trauma patients	10	…we work as a unit across the floor and help each other out… (Nurse)
Authority and support from leadership figures is important in the current and future care of trauma patients	8	At a cost of repetition, [anesthesiologist], [Emergency Room consultant], and [trauma surgeon] I believe are the leaders who are driving this forward. And we will be swinging on their tail, as they say. (Consultant)
Knowing your colleagues well and understanding their strengths and limitations in an established team improves patient care	7	[this city] is still fairly small, very large village, where everyone knows everyone, they all know me when I arrive on the scene… (Consultant)
We need to work together with national and regional health bodies and outside organizations when planning the transition to MTC	4	it will allow us to develop really, it provides a stimulus for us to develop good relationships with our, [local health boards], uh, to, uh, attract the activity from the [region of Scotland]. (Manager)
Emotions		
*No belief statements*		
Behavioral Regulation		
*No belief statements*		

#### Social Influences (Four belief statements)

Frequent belief statements included the importance of good teamwork and good leadership, and working in a well-established team. A less frequent belief describes the importance of working with national and regional organizations in order to facilitate the transition to a MTC.

#### Skills (Four belief statements)

Frequent beliefs in this domain included sufficient levels of non-technical skills like communication and teamwork and a desire to learn trauma skills from others, such as visiting other MTCs. Participants believed that managing trauma patients is routine for them, and that their skill levels would improve further if the hospital became an established MTC.

#### Beliefs about consequences (Three belief statements)

Participants described a highly frequent belief statement that becoming a trauma center would have a positive impact on patient care, with more resources, more patients, better recruitment and trauma being a higher priority. Less frequent beliefs included that staff morale would be improved by becoming a MTC, and that having a coordinated hospital trauma response would make patient outcomes better.

#### Motivation and goals (Three belief statements)

All participants expressed the belief statement that they were motivated to be involved in the transition to a MTC. Moderately frequent beliefs included participants wanting to deliver their best care and improve it, and that goals related to trauma should be a high priority.

#### Social/professional role and identity (Three belief statements)

A highly frequent belief described participants’ desire to play a role in the transition to a MTC. Moderately frequent beliefs included participants wanting to be involved in the initial assessment and resuscitation of the trauma patient, and a belief that someone should lead and coordinate the care of multiple-injured patients though the hospital.

#### Knowledge (Two belief statements)

Participants described a highly frequent belief that they keep up to date with the latest evidence in trauma care, and a moderately frequent belief that they have the knowledge required to manage trauma patients.

#### Beliefs about capabilities (One belief statement)

Participants described a highly frequent belief that they are capable of improving the care they provide in the hospital and changing the culture in order to become a MTC, overcoming difficulties in the process.

#### Optimism, reinforcement, intentions, memory attention and decision processes, environmental context and resources, emotions, behavioral regulation

No belief statements classified as important enablers were mapped to these domains.

### Key barriers *OR* enablers

Twenty-four belief statements were classified as either a barrier or enabler (Table 4). These either had a condition that if met, or not met, may act either as a barrier or enabler, or were discordant belief statements with opposing beliefs amongst participants. Most belief statements were either reported in both positive and negative forms within the same interview, or were conditional rather than discordant beliefs. Therefore it was impossible to report individual participant ‘positive’ and ‘negative’ frequencies for each belief statement.

**Table 4:** Summary of belief statements classified as barriers *OR* enablers

Belief statement (n = number of participants expressing the belief)	n	Example quote
Knowledge		
There are (no) credible guidelines or algorithms for trauma patients at the hospital which improve patient care	10	I think they’re still work in progress. (Consultant)
Skills		
*No belief statements*		
Social/Professional Role and Identity		
I do (not) see trauma as a large part of my role	10	A huge part of my role, it’s exactly why I chose to do emergency medicine, it’s exactly what interests me, um, and without the possibility of seeing major trauma I probably wouldn’t choose to do emergency medicine. (Registrar)
Management and politicians play a positive/negative role in steering the trauma service	8	I think, um, from management levels, I’m not sure how, I get a feeling there’s reluctance but I don’t know if that is, is true or not. (Registrar)
Beliefs about Capabilities		
I am (not) capable of aspects of my own role in looking after trauma patients	9	I think I would have the skills, yeah. And confidence, and that confidence will only get better the more you see. (Registrar)
We do (not) provide good care as a hospital for the current caseload of trauma patients at present	9	…that’s [patients remaining in resus for prolonged periods of time], actually that’s, for me that’s a marker of a system that isn’t working, that isn’t getting the patient to their care, definitive care location. Um, and having, um, system-wide ownership of that patient. (Consultant)
My colleagues are (not) capable of adequately providing trauma care	7	I am very confident of my orthopedic colleagues, because we have a very good orthopedic department, and uh, the reputation of their trauma training is quite good. So I have no hesitation about, uh, my orthopedic colleagues with whom I work. (Consultant)
Optimism		
I’m optimistic/pessimistic about the changes being made and the role of major trauma at the hospital	10	I, I’m highly confident that we can. I’m highly confident that we could do it. (Manager)
My optimism/pessimism is conditional upon availability of necessary resources	4	…if we had everything that I’ve just described to you, plus the authority to make it happen, I reckon we could probably have it up and running by this time next year. (Consultant)
Beliefs about Consequences		
Becoming a trauma center would affect the effectiveness of myself, my colleagues or the hospital in a positive/negative manner	10	…well to a large extent, because not only would we meet the needs of patients who are suffering from major trauma, much more effectively, uh, but we’d, I believe that developing [this hospital] into a major trauma service will improve the efficiency, the clinical efficiency of the hospital as a whole. (Manager)
Becoming a MTC would (not) influence patient views of their care	10	Um, I hope so. I think, uh, I guess it’s interesting, I mean there’s so much of this on telly now. I hope the public start asking questions about, you know, how we organize it and…I think their expectations ought to be a little bit different now. (Consultant)
Reinforcement		
I am (not) aware of any material rewards for becoming a trauma center	10	Hopefully if we were a major trauma center we’d get a bit more funding as well to, to expand our roles. (Registrar)
Intentions		
I am (not) planning to change the way either I or the hospital care for trauma patients	9	We are just now, we’ve decided to do this slightly differently, so we’re gonna have a group of four, we should come back to this action, we’re gonna have a group of four and we’re gonna meet probably every six to eight weeks, and that is one from ED, one from anesthetics, one’s from orthopedics and one from general surgery. Um, a, to look at the cases that are highlighted, cos although we're currently doing it, it‘s not being done with all four specialties, so we’re, that’s starting next month. Uh, to highlight those again, to take to the multi-disciplinary meetings. (Consultant)
We are (not) intending to contribute more towards resources and staffing to support trauma care and the transition to a MTC	6	We do have a, uh, another proposal for a coordinated hospital trauma response, and it’s good to go, and it’s gonna have to happen because of, um, changes that are happening within the emergency department, it is going to have to happen, um, so the timing around major trauma center is, is good from that perspective. (Consultant)
Motivation and Goals		
I do (not) know about goals for developing trauma services	10	…then I guess having that, if we all know what the, what our timelines are…that becomes the end point…it then becomes just A to Z, and it within the timescale. (Manager)
Achieving goals depends on the motivation of those involved, which is positive/negative	10	It’s gonna be hard work to move the agenda forward unless they all realise how important a trauma agenda is for the whole of [local health board] and the whole of [this part of Scotland], not only trauma patients, because otherwise we’ll be a [district general hospital]. (Consultant)
Departments and individuals have a high/low motivation for trauma care	8	Yeah, timeliness, attendance…essentially it was because the leadership of surgery didn’t buy into this as a concept. And that’s still the position. (Consultant)
Our service is affected positively/negatively by targets and goals imposed from government level	7	Whereas the vast majority of targets which are used as a stick if you like, to beat, a, uh, NHS board with, or indeed to allocate reward, are based upon elective waiting lists, rather than outcomes, and specifically outcomes of unscheduled care, which I think are, uh, very much the poor cousin. (Consultant)
Memory, Attention and Decision Processes		
*No belief statements*		
Environmental Context and Resources		
The hospital’s current trauma care and the transition to a MTC is affected by – and affects – the surrounding environment in a positive/negative manner	10	…because that’s essential, because we require the, uh, activity from the, uh, the major, from the [local health boards], um, to come to [this hospital], because we will always be marginal in terms of activity, uh, in relation to major trauma. (Manager)
The organizational culture at the hospital is (not) supportive and geared towards performance improvement	9	The greatest strength we have is that a very, very personal and not very formal or bureaucratic approach to team working. We can go to any colleagues without formal appointment and going through a secretary and this and that. And just knock the door and say, ’[xxx], can I discuss a case with you?‘ or, ’Can you help me?'. (Consultant)
Recruitment is difficult for the hospital, and may be made easier/harder by (not) becoming a MTC	7	If we didn’t have that, I think we’d lose a lot of folk…I probably would want to go to a major trauma center and work myself. (Registrar)
Social Influences		
My practice is (not) influenced by guidelines and protocols	8	I don’t know if they [guidelines] would make a difference or not, but if there was evidence that it would then I’d be all for it. (Registrar)
Emotions		
I do (not) get affected emotionally by providing major trauma care	10	But my prime frustration in managing major trauma is not making things happen that I know needed to, to happen, in terms of organizing a response from, from specialties within this hospital. (Consultant)
Emotions do (not) affect the care I provide	5	No. When you’re highly charged, I think you give the best care, and I wouldn't say there’s any time where I’ve been worried that my staff can't look after a patient. (Registrar)
Behavioral Regulation		
We (do not) currently have local and national auditing, monitoring and reporting procedures	10	I guess for the medical staff there’s the M and M meetings, but for us, as nurses there’s not really any formal recording (Nurse)

#### Motivation and goals (Four belief statements)

Participants described highly frequent belief statements around knowledge of goals to develop trauma services, with some being aware and others being unaware. Participants described variability in motivation for trauma care, with achievement of goals being dependent on this. They also described the influence on their service (either positive or negative) of targets imposed on the hospital from government level.

#### Beliefs about capabilities (Three belief statements)

Participants expressed highly frequent belief statements about their variable capability both as individuals (either themselves or their colleagues), and as a hospital, to provide good trauma care.

#### Environmental context and resources (Three belief statements)

A highly frequent belief statement described the relationship the hospital has with the surrounding environment and organizations, and the positive or negative effect this could have on the transition to MTC. Most participants described a belief about the organizational culture at the hospital, and how supportive (or unsupportive) it was. A moderately frequent belief statement described the recruitment challenges the hospital faces, and the influence that becoming a MTC could have on these.

#### Beliefs about consequences (Two belief statements)

Participants described mixed beliefs surrounding whether becoming a MTC would affect the effectiveness of many aspects of the hospital in a positive or negative manner, and whether becoming a MTC would affect patient views of their care or not.

#### Intentions (Two belief statements)

A highly frequent belief statement described whether participants were planning to change the way they manage trauma patients or not. A moderately frequent belief described whether participants were intending that more resources and staffing be put towards trauma care or not.

#### Emotions (Two belief statements)

Participants all described whether or not they get an emotional response in providing major trauma care. Half of them described whether or not this response affected the care they gave.

#### Optimism (Two belief statements)

All participants described a level of optimism or pessimism about the changes being made to trauma care at the hospital. There was a moderately frequent belief statement that this optimism or pessimism was conditional upon resource availability.

#### Social/professional role and identity (Two belief statements)

All participants described the extent to which they saw trauma as a part of their role, which was very variable. Most participants described the role of management and politicians in steering the trauma service, with mixed views as to whether this was a positive or negative influence.

#### Behavioral regulation (One belief statement)

There were highly frequent but mixed beliefs about the levels of audit and monitoring of trauma care at both local and national level.

#### Social influences (One belief statement)

Most participants discussed whether or not they were influenced by trauma guidelines and protocols at the hospital, with mixed beliefs.

#### Knowledge (One belief statement)

All participants discussed whether or not they were aware (or not) of trauma guidelines and protocols at the hospital, with mixed beliefs.

#### Reinforcement (One belief statement)

There were mixed beliefs regarding whether or not the hospital would get any material reward for becoming a trauma center.

### Skills, memory attention and decision processes

No belief statements classified as important barriers or enablers were mapped to these domains.

All 91 belief statements are presented online as Additional file 3, grouped by domain, alongside sample quotes.

## Discussion

This study identifies multiple barriers and enablers to implementing a MTC in a tertiary hospital using systematic and replicable theory-based methods. Identifying such beliefs can highlight potential targets for context-specific interventions to facilitate this complex transition. This is crucial for creating tailored implementation strategies needed to develop specialist units like MTCs and other acute care networks worldwide. This study is one step towards addressing the recently highlighted gap in theory-based implementation research in the context of emergency medicine [12].

There is increasing recognition of the global burden of traumatic injury from bodies including the World Health Assembly, World Bank and Lancet Global Surgery Commission, with a call for improved data collection and analysis as well as dissemination of knowledge, techniques and systems from regions with sophisticated clinical practices and health systems to other areas [32–34]. It will be important to optimize translation and implementation of techniques and health systems which are well-established in high-income countries to similar geographic regions with less mature healthcare systems or low-to-middle-income-countries with developing infrastructure and systems. Establishing a MTC within a trauma system is a good example of such a process.

### Informing the transition to MTC status

Resource availability was identified as an important barrier despite the hospital being a major tertiary center. Given current fiscal conditions, this unsurprising finding has also been identified in recent studies of surgical practice, such as implementing checklists and enhanced recovery programs [35–37]. Resource needs should be clearly and transparently identified, costed, and addressed. Distracting priorities – in particular, increasing numbers of patients attending Emergency Departments – is another issue receiving widespread attention [38]. Safeguarding the trauma response could be achieved by separating responsibility for trauma from the remainder of the emergency medicine workload, as practiced in many American trauma centers – but this would result in additional resource burden. Enhancing individual skills is key to developing institutional expertise, and could be addressed through local initiatives like simulation, courses, and facilitating fellowships in existing MTCs. Consideration should also be given to establishing dedicated performance improvement programs for trauma – although this would also need resources.

A key enabler was the belief that becoming an MTC would benefit both patients and organization. This belief should be reinforced, through engagement with workforce, to further harness and amplify widespread motivation to improve care. This will be vital in helping address the barriers identified. Motivated front-line staff, aware of problems surrounding trauma care delivery, should play a large role in addressing them.

Communication challenges are common in implementation studies [35–37]. Present findings reveal a degree of conflict between clinicians and managers, who each regarded engagement of the other as a barrier to transition, and of knowledge dispersal amongst staff, such as goals for becoming MTC being confined to senior management, who believed all others were also aware. Improving communication between roles, tiers and departments on all aspects of detailed planning, goals, budgeting, must occur in order for this transition to succeed.

### Strengths and limitations of using the TDF to facilitate service development

High-level service development is complex, and typically informed by various methods, from analysis of performance data, to expert opinion. This study demonstrates the TDF’s added value to such methods, by systematically identifying theory-based barriers and enablers. Whilst many findings – particularly those around organization and resource management – may have been expected, use of the TDF has identified important issues that were, intuitively, not felt to be problematic, such as optimism/pessimism amongst staff about the transition being conditional on meeting resource demands.

The complexity of behaviors studied posed many challenges to applying the TDF in this context. The ‘behavior’ of implementing a MTC is highly complex, comprising multiple, distinct sub-behaviors at individual (e.g. surgical skills), departmental (e.g. quality improvement programs) and organizational levels (e.g. hospital trauma team). This then presented analytical challenges. For example, it was sometimes difficult to decide which domain a belief fit into: Guidelines may be ‘social influences’, a ‘goal’, or even ‘behavioral regulation’ if used to solve a problem. Prior to this study, TDF has been applied predominantly at individual clinician level, rather than hospital service level, despite not being exclusively designed for this purpose. It has been suggested that the domains within the TDF that relate to organizational factors (e.g. ‘Environmental Context and Resources;’ ‘Social Influences’) may not be sufficiently thorough to enable a comprehensive investigation of barriers/enablers to change at an organizational level. It may be that other frameworks such as Consolidated Framework for Implementation Research [39] may be useful in this context, since they adopt an organizational focus and were designed primarily for examining barriers and enablers to change across multiple settings and levels within an organization. However, it has been conversely argued that the CFIR does not sufficiently elaborate on individual-level barriers/enablers to change. Therefore, future research could adopt the CFIR/TDF concurrently to develop a topic guide that more comprehensively investigates individual- and organizational-level barriers to change. The complimentary use of both frameworks has recently been applied in other clinical contexts, for example, to develop a topic guide to explore barriers/enablers to blood transfusion clinical staff responding to transfusion audit and feedback [17].

Although analysis was inherently prone to a degree of subjectivity, the high level of agreement between coders (quantified through *kappa* statistics) indicates minimal risk of subjective bias. This was a highly contextualized study aiming not only to identify targets for change in this specific hospital, but to illustrate a replicable, systematic method that could be applied in other hospitals to address similar questions, or to address implementation problems in other contexts. Qualitative research does not aim to identify readily generalizable, ‘universal’ findings, but rather to explore a topic in-depth and identify emerging, key context-specific themes, which could be explored in future research. Therefore the present findings may not be readily generalized beyond the context of the tertiary center where the study was conducted. Different hospitals and indeed different health systems will have different pressures and resource needs, with potentially different belief structures. Nonetheless, the generalizability of the present findings could be explored through TDF–based questionnaires based on these results, distributed in different hospitals. This approach has previously been applied to explore generalizability of TDF interview-based findings [16].

Lessons Learnt:

- Resource needs are a large concern despite the hospital already being a tertiary center. Optimism or pessimism about the transition is conditional on these being met.
- Distracting priorities abound within the currently overloaded Emergency Department.
- Skills could be developed through simulation or fellowships at established trauma centers, and a solid performance improvement program should be implemented.
- Engaging staff in the transition will be crucial to harnessing their high motivation for delivering good trauma care.
- Communication must be more transparent between roles and tiers since there are currently large gaps in goal awareness and miscommunications.

## Conclusions

This study used the TDF to systematically and replicably examine and identify targets to guide the complex change management processes in a tertiary center facing the challenge of transforming into a MTC. Despite being a tertiary center there were still major concerns about resourcing the transition, and a need was identified for improving specific trauma skills, along with establishing performance improvement processes. In common with other recent surgical implementation studies [35–37], communication problems existed throughout, and engaging all staff in a transparent development process will address this and harness their motivation for delivering high quality care. These findings can inform implementation techniques to facilitate the transition. Work is currently underway to map specific Behavioral Change Techniques to TDF domains, in order to provide guidance as to which techniques may work to specifically address barriers or enablers related to each TDF domain [40, 41]. TDF-based methods and interventions can then be applied to facilitate complex change management processes such as the development of cardiac arrest or stroke centers, or to development of MTCs in other settings with different cultural challenges.

### Key messages

- Implementing the transformation process of developing a tertiary center into a MTC has many barriers and enablers, including resource allocation, need for improved skills, need for establishing performance improvement programs, and poor communication.
- Establishing these barriers and enablers prior to implementation facilitates development of an intervention to improve this transition.
- This study uses a replicable, theory-based method using the TDF in order to elicit these barriers and enablers.
- This method could be applied to facilitate other complex developments or interventions such as developing specialist cardiac arrest or stroke centers, or MTCs in developing countries.

## Additional files

Additional file 1: Interview script. (DOCX 28 kb) Additional file 2: Table showing theme presence in each transcript. (DOCX 30 kb) Additional file 3: Complete Results Table, sorted by TDF Domain. (DOCX 50 kb)
