# Supplementary material for: What helps or hinders the transformation from a major tertiary center to a major trauma center? Identifying barriers and enablers using the Theoretical Domains Framework
Source: Scand J Trauma Resusc Emerg Med. 2016 Mar 12;24:30. doi: 10.1186/s13049-016-0226-3 (PMC4788933; doi:10.1186/s13049-016-0226-3)
Supplement: Additional file 1: — Interview script. (DOCX 28 kb) [file 13049_2016_226_MOESM1_ESM.docx]

**Additional File 1: Interview script.** (TDF domains highlighted in blue)

**[BEFORE INTERVIEW OFFICIALLY BEGINS]**

Thank you for taking the time to participate in our study. As you may know, we are interested in understanding the barriers and facilitators to changing a university teaching hospital into a major trauma centre.

Can I first double check that you have had a chance to review the information sheet and consent form? Do you have any questions about the study?

Do you agree to participate in this study knowing that you can withdraw at any point with no consequences to you?

If participant does not agree: End interview. “Thank you very much for your time. We will not contact you further about the study.”

**If participant agrees:**

Thank you for your participation. We will now begin the interview. It should take approximately 40 minutes. You may choose not to answer any question. You may provide brief answers or go into as much detail as you choose. There are no “correct” answers. We are interested in your perspectives as clinicians and experts in patient care.

With your permission, I’d like to audio-tape the interview.

To preserve confidentiality, please try not to identify any health care worker, patients or hospitals by name. If you do identify anyone by name, we will delete this information from the transcript before anyone else sees it.

**[OFFICIAL START OF INTERVIEW/ I.E. START OF AUDIO-RECORDING]**

**[*Part 1- Current/General Practice in Major Trauma Care]***

*This interview will be conducted in three main parts…In this first part I would like to discuss your general views and current role in major trauma care.*

1. **Can I begin by asking how long you have been working at [this hospital]?**
2. **And just briefly, what is your primary role in the hospital?**
3. **How many years have you been working in this role?**
4. **And just briefly, how much experience do you have in major trauma care?**
5. **To what extent do you see providing major trauma care as a part of your role?** *(Social/Professional Role and Identity)*
   1. *Initial phase of care of major trauma patients*
   2. *Ongoing hospital care of major trauma patients*
6. **To what extent do you think providing major trauma care is part of the role of your colleagues both within your department and in this hospital as a whole?** (*Social/Professional Role and Identity)*
   1. *To what extent do you think these colleagues provide major trauma care in the same way?*
7. **How many major trauma cases do you typically see?** (*Social/Professional Role and Identity)*
   1. *Is caring for them routine for you?*
8. **How easy or difficult do you find providing major trauma care?** *(Beliefs about Capabilities/Skills)*

**a)** *Are there any specific problems or difficulties you have encountered in providing major trauma care?*

1. **To what extent do you feel affected emotionally in any way by providing major trauma care?** *(Emotions)*
   1. e.g. *Stressful/ afraid/ anxious/ sad/ concerned*
   2. ***If yes,*** *does this impact the care you give to major trauma patients?*
2. **In general, to what extent do you feel you have the necessary skills or training to contribute to major trauma care?** *(Skills)*
   1. Have you had any formal training in major trauma care? ***If not,*** *what is lacking?*
   2. *What about non-technical skills (eg communication, planning , persuasion)?*
   3. *What about your colleagues in your department and this hospital in general? To what extent do you feel they have the necessary skills or training to provide major trauma care?* ***If not,*** *what is lacking?*
3. **Are you aware of any hospital guidelines, protocols, policies or algorithms that may be used in major trauma care?** *(Knowledge)*
   1. ***If yes:*** *Are you familiar with the content of these (i.e. guidelines/ protocols/ policies/ algorithms)?*
   2. *How credible do you think these guidelines/ protocols/ policies/ or algorithms are?*
   3. *To what extent would you say these guidelines influence your provision of major trauma care?*
4. **To what extent do the views or practice of other colleagues in the department or hospital influence the care you provide to major trauma patients?** *(Social Influences)*
   1. *Who influences?*
   2. *How?*
5. **In your opinion, is there a supportive environment in this hospital in which you can ask questions if you are unsure about aspects related to major trauma care?** *(Social Influences)*
6. **In general, how do you and/or your colleagues monitor the major trauma care you provide?** *(Behavioral Regulation)*
   1. *Who monitors*
   2. *What monitor*
   3. *When monitor*
   4. *How monitor*
   5. Are you aware of any hospital performance improvement processes in place for major trauma care?
      1. *If yes,* can you talk me through these?
   6. *Are you aware of any audits of major trauma care conducted by this hospital?*
      1. ***If yes,*** *who conducts audit?*
      2. *What is audited*
      3. *How often audited*
   7. *Do you submit data to a major trauma registry?*
7. ***Have you ever received feedback on the major trauma care you provide?*** *(Behavioral Regulation)*
   1. *Are there regular morbidity/case review/audit meetings?*
   2. ***If yes,*** *how often?*
   3. *Who attends?*
   4. *Do you typically attend? How regularly?*
   5. *Are the feedback and/or meetings and/or feedback useful or helpful? If yes, how so?*
8. **In general, what specialist resources do you think are required for you in your role to specifically provide major trauma care effectively?** *(Environmental Context and Resources)*
   1. *What Staffing is needed for your department?*
   2. *What specialist Equipment is needed for your department?*
   3. *What Physical set up is best (e.g. layout) for your department?*
   4. *To what extent would these requirements differ for your role in initial care or in ongoing major trauma care?*
   5. *To what extent do you feel this hospital already has sufficient levels of these resources available?* ***If not,*** *why not?*
9. **In general, are there any other competing activities, priorities or demands that require your attention which interfere with the provision of major trauma care?** *(Memory, Attention and Decision Processes)*
   1. ***If yes:*** *compared to providing major trauma care, where would you rank these in terms of priority?*
   2. *To what extent do you think these competing activities, priorities or demands would differ in an established major trauma centre?* ***If yes****, how so?*
   3. To what extent do you feel you have sufficient time to contribute to the care of major trauma patients?
      1. *Early care*
      2. *Ongoing care*
10. **Other than the potential transition to major trauma centre, are there any changes you are currently planning to make to either you or the hospital’s practice in major trauma care?** *(Behavioral Regulation/Intentions)*
    1. **If yes**- What? Who will implement change? When? How? (talk through)

**[*Part 2- Major Trauma Centres]***

Thank you for your input so far, I would like to now move onto the second part of this interview, where we will discuss care in major trauma centres, in general:

1. **In general, how would you describe a major trauma centre?** *(Knowledge)*
2. **What do you think is the evidence for major trauma centres?** *(Knowledge)*
   1. *How often do you keep up with new, emerging evidence for major trauma care?*
3. **To what extent would your current role in major trauma care be different in an established major trauma centre?** (*Social/Professional Role and Identity)*
4. **And to what extent would the role of your colleagues be different in an established major trauma centre?** (*Social/Professional Role and Identity)*
5. **To what extent do you feel you have the necessary skills or training to contribute to major trauma care in an established major trauma centre?** *(Skills)*
   1. ***If not,*** *what is lacking?*
   2. ***What about non-technical skills (eg communication, planning, persuasion)?***
   3. *And what about your colleagues or other clinicians in this hospital?* ***If not,*** *what is lacking?*
6. **What would you see as the benefits of becoming a major trauma centre?** *(Beliefs about Consequences)*
7. **What about the disadvantages of becoming a major trauma centre?** *(Beliefs about Consequences)*
8. ***To what extent do you think the benefits of becoming a major trauma centre outweigh the costs of becoming one?*** *(Beliefs about Consequences)*
9. **To what extent would you say your general views of major trauma centres are shared by your colleagues in this hospital?** *(Social Influences)*

**[Part 3- Changing a university teaching hospital into a major trauma centre]**

*Thank you for your input so far. In the third part of this interview I would like to specifically discuss with you your views on the process of changing this hospital into a major trauma centre.*

1. **Are you aware of the developing major trauma centre system in Scotland?** *(Knowledge)*
2. ***If yes:*** *Can you elaborate please?*
3. *Are you aware of what role this hospital will play in this developing trauma system?*
4. **In general, what do you think would need to change in order to change a university teaching hospital into a major trauma centre?** *(Knowledge)*
5. *What would need to change?*
6. *How complex would say the transition process is?*
7. *Approximately how long do you think the process takes?*
8. *To what extent do you think it would be necessary to trial or practice aspects of this transition first?*
9. *To what extent do you think the process of changing into a major trauma centre would be disruptive to current practice in anyway?*  *(Beliefs about Consequences)*
10. ***If so****, what can be done to minimize any disruption?*
11. *In general, what resources do you think would be needed to support the transition from a university teaching hospital to a major trauma centre? To what extent do you feel this hospital has sufficient levels of these resources available?* ***If not****, why not?* *(Environmental Context and Resources)*
12. **More specifically how confident would you say you are that this hospital could successfully change into a major trauma centre?** *(Beliefs about Capabilities)*
13. To what extent do you feel this hospital is ready for the transition? Please elaborate? *(Optimism/Beliefs about Capabilities)*
14. What would need to change in this hospital specifically to become a major trauma centre?
15. What would need to stay the same in this hospital?
16. **To what extent do you consider contributing to this hospital’s transition to a major trauma centre part of your role?** (*Social/Professional Role and Identity)*
17. **Compared with other tasks you have to do in your role, where would you rank contributing towards this hospital’s transition into a major trauma centre in terms of priority?** *(Memory, Attention and Decision Processes)*
18. **In your opinion, who else should be involved in changing this hospital into a major trauma centre?** (*Social/Professional Role and Identity)*
19. Are you aware of anyone who has been formally appointed to lead this transition process?
20. In your opinion, what additional internal or external support is needed from other types of colleagues to support this transition (e.g. managers, project coordinators, other similar roles)?
21. **What would facilitate this hospital’s transition into a major trauma centre?** *(Beliefs about Capabilities)*
22. What would interfere?
23. **Are you aware of any ways in which becoming a major trauma centre is encouraged or rewarded?** *(Reinforcement)*
24. *Externally? Internally? (Motivation and Goals)*
25. **To what extent do you feel there is any internal or external pressure to become a major trauma centre?** *(Social Influences)*
26. ***If yes,*** *please elaborate.*
27. **Are you aware of any goals that have been set for the changing of this hospital into a major trauma centre?** *(Motivation and Goals)*
    1. **If yes,** What are they? Who decided on these? Who disseminated to?
28. **Are you aware of any action plans that have been made for changing this hospital into a major trauma centre?** *(Behavioral Regulation/Intentions)*
    1. **If yes,** What are they? Who has decided these? Who disseminated to?
29. **Overall, how do you feel about this hospital becoming a major trauma centre?** *(Emotions)*
    1. *E.g. afraid, worried, anxious, excited, optimistic*
    2. ***If negative emotions:*** *what could be done to help reduce your [anxiety/ stress/ concerns/ fears/ etc]*
30. **Have you specifically discussed the transitioning of this hospital into a major trauma centre with any colleagues?** *(Social Influences)*
    1. **If yes,** with whom?
    2. Where? Was this at a specific meeting?
    3. **If yes,** who attended this meeting?
    4. How often have you discussed the transition to a major trauma centre?
31. **In your opinion, what do your colleagues think about this hospital becoming a major trauma centre?** *(Social Influences)*
32. *Is there currently any disagreement or conflict over the transition into a major trauma centre?*
33. **To what extent would changing this hospital to a major trauma centre enable you or your colleagues to better meet patient needs?** *(Social Influences)*
    1. *Would changing this hospital into a major trauma centre influence patient views of major trauma care?*
    2. **If yes,** how so?
    3. **If not,** why not?
34. **And lastly, to what extent do you feel committed to becoming a major trauma centre?** *(Motivation and Goals/Intentions)*

***a****. to what extent do you feel other colleagues feel committed to becoming a trauma centre?*
