# Supplementary material for: What helps or hinders the transformation from a major tertiary center to a major trauma center? Identifying barriers and enablers using the Theoretical Domains Framework
Source: Scand J Trauma Resusc Emerg Med. 2016 Mar 12;24:30. doi: 10.1186/s13049-016-0226-3 (PMC4788933; doi:10.1186/s13049-016-0226-3)
Supplement: Additional file 2: — Table showing theme presence in each transcript. (DOCX 30 kb) [file 13049_2016_226_MOESM2_ESM.docx]

Additional File 2: Table showing theme presence in each transcript, with data saturation achieved by Participant 11.

| **Theme** | **1** | **2** | **3** | **4** | **5** | **6** | **7** | **8** | **9** | **10** | **11** |
| --- | --- | --- | --- | --- | --- | --- | --- | --- | --- | --- | --- |
| I do not attend local governance meetings |  |  |  |  | x | x |  |  |  |  |  |
| We (do not) currently have local and national auditing, monitoring and reporting procedures | x | x | x | x | x | x | x | x | x | x | x |
| Guidelines and protocols can reduce the impact of lack of experience in junior staff | x |  |  |  |  |  |  |  |  |  |  |
| SOPs keep practice consistent |  | x |  |  |  |  |  |  |  |  |  |
| A good governance model will spread throughout the organization |  | x |  |  |  |  |  |  |  |  | x |
| Ongoing site development plans impact on solving our current problems |  |  |  |  | x |  |  |  |  |  |  |
| Governance processes take a lot of time |  |  |  |  | x |  | x |  |  |  |  |
| A transition to a trauma center should be carefully planned | x |  |  |  |  |  |  |  |  |  | x |
| We are planning to solve our staffing problems to facilitate the transition to MTC |  |  | x |  | x |  |  |  |  | x |  |
| We are planning to use the process of becoming a MTC to solve problems in other areas of the hospital |  |  |  |  |  |  |  |  | x |  |  |
| We are planning to reorganize the hospital to facilitate the transition to MTC |  |  | x |  |  |  |  |  |  | x |  |
| Sometimes I require others to help me perform parts of my role in looking after major trauma patients. | x | x | x | x |  |  | x | x |  |  | x |
| We are capable of improving our practice and changing our culture to become a MTC, though it may be difficult in places | x | x | x | x | x | x | x | x | x |  | x |
| I am (not) capable of aspects of my own role in looking after trauma patients | x | x |  | x | x | x | x | x | x | x | x |
| My colleagues are (not) capable of adequately providing trauma care. | x | x |  | x | x | x | x |  |  | x | x |
| We do (not) provide good care as a hospital for the current caseload of trauma patients at present | x | x | x | x | x | x | x | x |  | x | x |
| I find it easy to/struggle to work with SOPs and guidelines |  | x |  |  |  |  |  | x |  |  |  |
| You can deviate from guidelines if you’re capable. |  | x |  |  |  |  |  |  |  |  |  |
| A co-ordinated approach to efficiently meeting and treating trauma patients would make outcomes better | x |  | x |  |  |  | x | x |  |  | x |
| Becoming a trauma center would improve staff morale |  | x |  |  |  |  | x | x |  |  |  |
| Becoming a trauma center would affect the effectiveness of myself, my colleagues or the hospital in a positive/negative manner | x | x | x | x | x | x | x | x | x | x | x |
| Becoming a MTC would/not influence patient views of their care | x | x | x | x | x | x | x | x | x | x | x |
| Becoming a trauma center would lead to better patient care (more resources, higher priority, more patients, better recruitment) | x | x | x | x | x | x | x | x | x | x | x |
| Guidelines, audit and regulation make outcomes better |  | x |  |  |  |  | x | x |  |  |  |
| I do (not) get affected emotionally by providing major trauma care. | x | x | x | x | x | x | x | x | x | x | x |
| Emotions do (not) affect the care I provide. |  | x |  | x | x | x | x |  |  |  | x |
| Debriefing and other coping strategies are important in trauma care. | x | x |  |  |  |  |  |  |  |  |  |
| We (do not) currently have enough levels of resources to provide good trauma care. | x | x | x | x | x | x | x | x | x | x | x |
| Substantially more staffing and resources, and maintenance of those already in place, would be required to effectively become a MTC. | x | x | x | x | x | x | x | x | x | x | x |
| The hospital is not organized in the optimum manner for trauma care and a reorganization would improve this. | x | x | x | x | x | x | x | x | x | x | x |
| It’s not clear how much becoming a MTC will cost or benefit, and funding it may be difficult. | x | x | x | x |  | x |  | x | x | x | x |
| The organizational culture at this hospital is (not) supportive and geared towards performance improvement | x | x |  | x | x | x | x | x | x | x | x |
| This hospital’s current trauma care and the transition to a MTC is affected by – and affects – the surrounding environment in a positive/negative manner | x | x | x | x | x | x | x | x | x | x | x |
| Recruitment is difficult for this hospital, and may be made easier/harder by (not) becoming a MTC | x | x | x |  |  | x | x |  | x | x |  |
| I am (not) planning to change the way either I or the hospital care for trauma patients | x | x | x |  | x | x | x | x | x | x | x |
| We are (not) intending to contribute more towards resources and staffing to support trauma care and the transition to a MTC. |  | x | x | x | x | x |  |  | x |  |  |
| I’m intending to discuss the trauma service with my colleagues |  |  |  |  | x |  |  |  |  |  |  |
| Others have variable or limited knowledge of trauma | x | x | x |  |  |  |  |  | x | x | x |
| I do not know what the resource requirements are for current trauma care or for becoming a MTC |  |  | x | x |  |  |  |  | x | x | x |
| I know about trauma care and how to manage trauma patients | x | x | x | x |  |  | x |  |  |  | x |
| There are (no) credible guidelines or algorithms for trauma patients at this hospital which improve patient care | x | x | x | x | x | x | x | x | x | x | x |
| I keep up to date with evidence for major trauma care | x | x | x | x | x |  | x | x | x | x |  |
| Knowledge about the challenges of trauma care is improving |  | x |  |  |  |  |  |  |  |  |  |
| Knowledge of the working environment is important |  |  |  |  | x |  | x |  |  |  |  |
| Information and data is important to my role in trauma care |  |  | x |  |  |  | x |  |  | x |  |
| There are numerous potential distracting priorities at the same time as trauma that do not allow me to do my job and impact on patient care |  | x | x | x | x | x | x | x | x | x | x |
| Being able to manage uncertainty and make decisions is important to trauma care | x |  |  |  |  |  |  | x |  | x |  |
| Decision making is affected by some services not being available 24/7 |  | x |  |  |  |  |  |  |  |  | x |
| Guidelines help decision making |  |  | x |  |  |  |  | x |  |  |  |
| Separating the medical and trauma presenting as unscheduled care will allow appropriate teams to deal with them |  |  |  |  |  |  |  |  |  | x |  |
| We should aim to deliver our best care and improve on it | x |  |  | x | x |  | x |  | x |  |  |
| Goals related to trauma care should be a high priority | x | x | x | x |  |  | x |  | x |  | x |
| I do (not) know about goals for developing trauma services | x | x | x | x | x | x | x | x | x | x | x |
| Achieving goals depends on the motivation of those involved, which is positive/negative | x | x | x | x | x | x | x | x | x | x | x |
| Our service is affected positively/negatively by targets and goals imposed from government level | x | x | x | x |  |  | x |  | x | x | x |
| Departments and individuals have a high/low motivation for trauma care | x | x | x | x |  |  | x | x | x | x | x |
| I am motivated to be involved in the transition to MTC | x | x | x | x | x | x | x | x | x | x |  |
| I’m optimistic/pessimistic about the changes being made and the role of major trauma at the hospital | x | x | x | x | x | x | x | x | x | x | x |
| My optimism/pessimism is conditional upon availability of necessary resources |  | x |  |  |  |  |  | x | x | x | x |
| I am (not) aware of any material rewards for becoming a trauma center. | x | x | x | x | x | x | x | x | x | x | x |
| Elective work is rewarded more than emergency work. | x |  |  |  |  |  |  |  |  |  |  |
| There is no formal reward for activities such as training. |  |  |  |  |  | x |  |  |  |  |  |
| In general, there are (not) sufficient levels of the necessary technical skills at this hospital to provide major trauma care | x | x | x | x | x | x | x | x | x | x | x |
| There are (not) sufficient amounts of teaching and training in trauma care at [this hospital] |  | x | x |  | x | x | x | x | x | x | x |
| Maintaining skills is important as well as developing them |  | x |  | x |  |  |  | x |  | x | x |
| We can improve our care by learning skills from others both within and outwith trauma. |  | x | x | x | x |  |  | x | x | x | x |
| Skills in major trauma care would be better if this hospital were to become an established MTC. |  | x | x |  | x |  | x | x | x | x | x |
| Managing trauma patients is routine | x | x | x |  |  | x |  | x | x |  |  |
| There are sufficient levels of the necessary non-technical skills at this hospital to provide major trauma care | x | x | x | x | x | x | x |  |  | x | x |
| If you have experience and skills there is less need for protocols and guidelines –but these help if you don’t | x |  |  |  |  |  |  | x |  |  |  |
| It is important not to lose skills from elsewhere by focusing on trauma |  |  |  |  |  |  |  |  |  | x | x |
| Teaching and training improves performance |  | x |  |  |  | x |  |  |  |  | x |
| It is important to train with the people you work with |  | x |  |  |  |  |  |  |  |  |  |
| My skillset is used in stressful and emotive situations |  | x |  |  |  |  |  |  |  |  |  |
| Trialling or practising aspects of becoming a MTC would be helpful |  |  |  | x | x | x |  |  |  | x | x |
| Management, nursing and medical staff do not work well together at present |  | x |  |  | x |  |  | x |  |  |  |
| There is variation amongst the views of myself and my colleagues about the transition to a MTC. | x | x | x | x | x | x |  | x | x | x | x |
| Authority and support from leadership figures is important in the current and future care of trauma patients | x | x | x | x |  |  | x | x | x | x | x |
| Knowing your colleagues well and understanding their strengths and limitations in an established team improves patient care. |  | x | x | x |  |  | x | x | x | x | x |
| We need to work together with national and regional health bodies and outside organizations when planning the transition to MTC. |  | x | x |  |  |  |  |  | x | x | x |
| My practice is (not) influenced by guidelines and protocols |  | x | x | x | x | x | x | x | x |  | x |
| Good teamwork is important to the current and future care of trauma patients | x | x | x | x | x | x | x | x | x | x | x |
| I practice in the same way as my colleagues and peers |  |  | x | x | x |  |  | x |  |  | x |
| I should play a role in the initial assessment and resuscitation of the patient | x | x | x | x |  |  | x | x |  |  | x |
| Someone should lead and coordinate the care of trauma patients through hospital |  |  | x | x |  | x | x | x |  |  |  |
| I should play a role in the transition to major trauma center | x | x | x | x | x |  |  | x | x | x |  |
| Management and politicians play a positive/negative role in steering the trauma service. | x | x | x | x | x | x |  | x | x |  |  |
| I do (not) see trauma as a large part of my role | x | x | x | x | x | x | x | x | x | x | x |
| I do not fully appreciate the extent of others’ roles | x |  |  |  | x |  |  | x |  |  |  |
| I play a role in local and national boards/organizations |  | x |  |  |  |  |  |  |  |  |  |
| I currently play a role in improving the service the hospital provides to trauma patients |  |  | x |  | x |  |  |  |  | x |  |
