# Supplementary material for: What helps or hinders the transformation from a major tertiary center to a major trauma center? Identifying barriers and enablers using the Theoretical Domains Framework
Source: Scand J Trauma Resusc Emerg Med. 2016 Mar 12;24:30. doi: 10.1186/s13049-016-0226-3 (PMC4788933; doi:10.1186/s13049-016-0226-3)
Supplement: Additional file 3: — Complete Results Table, sorted by TDF Domain. (DOCX 50 kb) [file 13049_2016_226_MOESM3_ESM.docx]

Additional File 3: Complete Results Table, sorted by TDF Domain.

| **TDF Domain** | **Identified Theme** | **Frequency (*n* transcripts)** | **Interviewees (Roles, *n*)** | **Sample Quote** |
| --- | --- | --- | --- | --- |
| **Behavioral Regulation** | I do not attend local governance meetings | 2 | Nurse (2) | ...I used to attend when I could, our M and M meeting you know, with the four consultants, but at the minute there's like sixteen-odd consultants and growing more and more by the day, so, it's difficult to attend and be part of that group and understand the, what's coming back from that morbidity, you know, um, conversations that they have... (Nurse) |
|  | We (do not) currently have local and national auditing, monitoring and reporting procedures | 10 | All | I guess for the medical staff there's the M and M meetings, but for us, as nurses there's not really any formal recording (Nurse)  All cases that come through, um, critical care, are audited via a, a national audit system, in terms of the outcomes. Um, that though primarily looks at the outcomes of mortality. (Consultant)  None that I know if in the hospital. Um, I, I would say not in the department either if I'm really honest , [are there any performance improvement programs?] (Consultant) |
|  | Guidelines and protocols can reduce the impact of lack of experience in junior staff | 1 | Consultant | I think certainly when more junior members of the team are involved I certainly think a guideline at the very least would be very helpful in allowing consistent practice, and at the best level. (Consultant) |
|  | SOPs keep practice consistent | 1 | Consultant | if I just phoned blood bank and say, 'Okay, I've got someone here who's bleeding out, just send me everything you've got,' some poor lass or guy at the other end of the phone is gonna get themselves into a twangle and panic, whereas if I say, 'Okay, I'd like to activate the major hemorrhage protocol please,' they can pull out a laminated sheet or something, whatever or their computer screen, and follow what they have to do, who they have to contact. (Consultant) |
|  | A good governance model will spread throughout the organization | 1 | Consultant | I'm a strong believer in hub and spoke effect. So if you've got a very strong hub relating to very strong integrated and meaningful governance processes, then that'll filter down the spokes. (Consultant) |
|  | Ongoing site development plans impact on solving our current problems | 1 | Nurse | And, you know, I have risk-assessed it and it has, it has um, gone, you know, to my manager and upwards, but because we are moving, there'd be no plans to put extra oxygen points in. (Nurse) |
|  | Governance processes take a lot of time | 2 | Registrar, Nurse | ...and I think what takes up a large part of our time at the minute is, healthcare environment work and inspections and audits surrounding that. (Nurse) |
|  | A transition to a trauma center should be carefully planned | 1 | Consultant | I think any transition, um, should be undertaken as part of a PDSA cycle, so, um, any small change, or any large changes indeed, should be consistently planned, um, undertaken, studied and then, uh, make an assessment of what other changes need to be undertake, need to be made. So it should basically be a consistent, um, approach that we are always evaluating what has happened, and always undertaking change to constantly improve. (Consultant) |
|  | We are planning to solve our staffing problems to facilitate the transition to MTC | 3 | Consultant, Manager, Nurse | I do think though that there are a number of attractions that we can use professionally to enhance recruitment. (Manager) |
|  | We are planning to use the process of becoming a MTC to solve problems in other areas of the hospital | 1 | Manager | major trauma is given a very high priority. Um, because it's a national priority, uh, but as I said earlier, because we have a belief that if we develop uh, major, [this hospital] as a major trauma center, uh, then it will help us to, um, fix a number of other issues. Um, you know, which will help to make the hospital, uh, operate more effectively. (Manager) |
|  | We are planning to reorganize the hospital to facilitate the transition to MTC | 2 | Consultant, Manager | We, we're trying to define our role as, because of our staffing crisis, we're trying to redefine our role as resuscitation of people with life and limb-threatening illness and injury. (Consultant) |

| **TDF Domain** | **Identified Theme** | **Frequency (*n* transcripts)** | **Interviewees (Roles, *n*)** | **Sample Quote** |
| --- | --- | --- | --- | --- |
| **Belief in Capabilities** | Sometimes I require others to help me perform parts of my role in looking after major trauma patients. | 6 | Consultant (4) Registrar (2) | ...one individual I think will never have either the skills or the ability to multi-task sufficiently to deal with all aspects of it, so, um, I can deal with, um, a given role, but the big thing is getting people with different skill sets involved… (Consultant) |
|  | We are capable of improving our practice and changing our culture to become a MTC, though it may be difficult in places | 9 | Consultant (4), Registrar (2), Nurse (2), Manager | Well I think the institution of an appropriate group trauma call system. I don't think that would take very long to, to, um, plan and implement, decide who you need, and then simply get a standard call system for that (Consultant) |
|  | I am (not) capable of aspects of my own role in looking after trauma patients | 9 | Consultant (3), Registrar (2), Manager (2), Nurse (2) | ...it's been difficult, uh, and, uh, it continues to be a little bit difficult, because I have to understand, uh, major trauma and what the implications will be for [this hospital] and uh, [local health board] and [local area] (Manager)  I think I would have the skills, yeah. And confidence, and that confidence will only get better the more you see. (Registrar) |
|  | My colleagues are (not) capable of adequately providing trauma care. | 7 | Consultant (3), Registrar, Manager, Nurse (2) | I am very confident of my orthopedic colleagues, because we have a very good orthopedic department, and uh, the reputation of their trauma training is quite good. So I have no hesitation about, uh, my orthopedic colleagues with whom I work. (Consultant)  Um, well quite a lot, because like some of them are newly qualified, new into the NHS, so I mean the skills that they're needing are, are basic moving and handling care, care of a post-operative patient, care of surgical site wounds, care of, you know, everything really. (Nurse) |
|  | We do (not) provide good care as a hospital for the current caseload of trauma patients at present | 9 | Consultant (4), Registrar (2), Manager, Nurse (2) | But in general I have seen the patients I (inaudible) last year or two where I was a part of the team, I only say that this is a tribute to the NHS, and more so to the [local] trauma service, in whatever the form and shape it exists now, that at least two of the patients who I felt would not have made it have done extremely well, and not only them, all the treating members are happy with the outcome and the patients and the family are very grateful, so I would say in general the level of care we provide even currently is good. (Consultant)  ...that's [patients remaining in resus for prolonged periods of time], actually that's, for me that’s a marker of a system that isn’t working, that isn't getting the patient to their care, definitive care location. Um, and having, um, system-wide ownership of that patient. (Consultant) |
|  | I find it easy to/struggle to work with SOPs and guidelines | 2 | Consultant, Registrar | I think it's, in terms of going on the ATLS course, I think there's protocol, um, driven in that sense, so in the acute phase, um, I think that's, that's straightforward to some degree. (Registrar) |
|  | You can deviate from guidelines if you’re capable. | 1 | Consultant | Unfortunately a lot of people that deviate from guidelines are the people that should never deviate from guidelines. (Consultant) |

| **TDF Domain** | **Identified Theme** | **Frequency (*n* transcripts)** | **Interviewees (Roles, *n*)** | **Sample Quote** |
| --- | --- | --- | --- | --- |
| **Belief in Consequences** | A co-ordinated approach to efficiently meeting and treating trauma patients would make outcomes better | 4 | Consultant (2), Registrar (2) | I think that would be really useful because a lot of time is spent looking to see what bleep number is this, this and this, and that's very time-consuming. (Registrar) |
|  | Becoming a trauma center would improve staff morale | 3 | Consultant, Registrar (2) | it will boost the morale of the staff employed here. They feel that they are doing something important, they feel valued. They will be able to work as a team, which will be further boost to their morale. (Consultant) |
|  | Becoming a trauma center would affect the effectiveness of myself, my colleagues or the hospital in a positive/negative manner | 10 | All | ...well to a large extent, because not only would we meet the needs of patients who are suffering from major trauma, much more effectively, uh, but we'd, I believe that developing [this hospital] into a major trauma service will improve the efficiency, the clinical efficiency of the hospital as a whole. (Manager)  More bureaucracy maybe. Yeah, more paperwork. (Consultant) |
|  | Becoming a MTC would/not influence patient views of their care | 10 | All | Um, I hope so. I think, uh, I guess it's interesting, I mean there's so much of this on telly now. I hope the public start asking questions about, you know, how we organize it and...I think their expectations ought to be a little bit different now. (Consultant)  No. [I don't think becoming an MTC would influence patient views] (Consultant) |
|  | Becoming a trauma center would lead to better patient care (more resources, higher priority, more patients, better recruitment) | 10 | All | I think the benefits are that we, that we can, uh, build an infrastructure and an image around it which becomes attractive, um, to, to recruiting the best staff we can. So there's a good reason to come here, because we're a major trauma center. (Manager) |
|  | Guidelines, audit and regulation make outcomes better | 3 | Consultant, Registrar (2) | ...we obviously get audited with the STAG data, so we always know what requirements that we need to have, to make trauma outcomes better. (Registrar) |
|  |  |  |  |  |
| **TDF Domain** | **Identified Theme** | **Frequency (*n* transcripts)** | **Interviewees (Roles, *n*)** | **Sample Quote** |
| **Emotions** | I do (not) get affected emotionally by providing major trauma care. | 10 | All | I enjoy it, to a degree, that's maybe a bit sick. (Registrar)  But my prime frustration in managing major trauma is not making things happen that I know needed to, to happen, in terms of organizing a response from, from specialties within this hospital. (Consultant)  Um, usually [I get affected] very little. (Consultant) |
|  | Emotions do (not) affect the care I provide. | 5 | Consultant (2), Registrar, Nurse (2) | No. When you're highly charged, I think you give the best care, and I wouldn't say there's any time where I've been worried that my staff can't look after a patient. (Registrar)  I don't, I don't think it does, it shouldn't. It shouldn't. I don't think personally it does with me, I think that's something that you take home with you rather than, (inaudible) you wouldn't certainly hope that a patient would see that you were affected by that. (Nurse) |
|  | Debriefing and other coping strategies are important in trauma care. | 2 | Consultant (2) | As I said, I've developed my own strategies to mitigate for the fact that I'm never debriefed. And I often use that sort of thing as my own way to debrief myself. And to, as I said before, reflect and improve my practice. (Consultant) |

| **TDF Domain** | **Identified Theme** | **Frequency (*n* transcripts)** | **Interviewees (Roles, *n*)** | **Sample Quote** |
| --- | --- | --- | --- | --- |
| **Environmental Context and Resources** | We (do not) currently have enough levels of resources to provide good trauma care. | 10 | All | A lot of our patients, the physio and OT service, as I said, it's priority of who's getting, you know, seen, rather than everybody who should be seen is seen. (Nurse)  Um, I don't know, I know the consultants have been in discussions about how many they predict they need to provide adequate care, and I can't remember what the shortfall is, but they're nowhere near what they need to run the emergency department here (Registrar)  I think certainly all the specialities are on site, which is a good, um, point. (Registrar) |
|  | Substantially more staffing and resources, and maintenance of those already in place, would be required to effectively become a MTC. | 10 | All | We would need to retain the speciality surgical services, such as cardiothoracic, such as neurosurgery, such as vascular… (Consultant)  I'm pretty sure that, uh, radiology for example requires additional resources (Manager) |
|  | The hospital is not organized in the optimum manner for trauma care and a reorganization would improve this. | 10 | All | I think that would be better if we could change the attitudes and have a, uh, arrest bleep or trauma bleep that we can go through switch and alert folk. (Registrar)  Similarly, um, the time of transit to the operating theatres is also, um, something of an issue, so again, um, the geographical positioning of those two is not ideal for that to happen (Consultant)  ...ways of looking at how many people need to be on a trauma rota, so I don't expect every general surgeon to want to do trauma, um, but if they are happy to facilitate a reasonable number to be on a rota to give that kind of level of response... (Consultant) |
|  | It’s not clear how much becoming a MTC will cost or benefit, and funding it may be difficult. | 8 | Consultant (4), Manager (2), Registrar, Nurse | If there are finite resources, and infinite demands, then somebody will have to make some compromises somewhere. And that's what the managers and the financiers will have to look into. (Consultant) |
|  | The organizational culture at this hospital is (not) supportive and geared towards performance improvement | 9 | Consultant (3), Registrar (2), Manager (2), Nurse (2) | The greatest strength we have is that a very, very personal and not very formal or bureaucratic approach to team working. We can go to any colleagues without formal appointment and going through a secretary and this and that. And just knock the door and say, '[xxx], can I discuss a case with you?' or, 'Can you help me? '. (Consultant)  I think the stumbling block, previously we were very fortunate with our acute general manager who was fairly senior but then the stumbling block came at the level of the clinical lead for the acute sector and the medical director, and then the board and the chief executive officer. (Consultant) |
|  | This hospital’s current trauma care and the transition to a MTC is affected by – and affects – the surrounding environment in a positive/negative manner | 10 | All | ...to, um, enhance our position within, you know the oil and gas sector, that we're uh, obviously sitting very close to, and these oil and gas are now highly regulated, in terms of health and safety, and we could be playing quite an important role in supporting them on that too. (Manager)  But we are in a unique position, because we have [patients from wide surrounding area] coming to us, and we also look after [distant islands], with occasional patients even from the [islands further afield], even though they normally go to [other large city]. I'm talking about neurosurgical workload. (Consultant)  ...because that's essential, because we require the, uh, activity from the, uh, the major, from the [local health boards], um, to come to [this hospital], because we will always be marginal in terms of activity, uh, in relation to major trauma. (Manager)  We seem to have an inordinately high amount of major trauma up here. Um, lots of road traffic collisions, but as, on Friday, um, farming accidents, industrial accidents. (Consultant) |
|  | Recruitment is difficult for this hospital, and may be made easier/harder by (not) becoming a MTC | 7 | Consultant (3), Registrar, Manager (2), Nurse | Well I think it would encourage people to want to come and work here. We have trouble with recruitment up here in [this hospital], um, recruiting people to the nursing side of things, due to the high, you know, expense of living up here, but if you had an acute trauma center it might be attractive to people coming in and help with recruitment and, and getting a high calibre of staff. (Nurse)  If we didn't have that, I think we'd lose a lot of folk...I probably would want to go to a major trauma center and work myself. (Registrar) |

| **TDF Domain** | **Identified Theme** | **Frequency (*n* transcripts)** | **Interviewees (Roles, *n*)** | **Sample Quote** |
| --- | --- | --- | --- | --- |
| **Intentions** | I am (not) planning to change the way either I or the hospital care for trauma patients | 9 | Consultant (3), Registrar (2), Manager (2), Nurse (2) | Not really. [planning to make any other changes] (Manager)  We are just now, we've decided to do this slightly differently, so we're gonna have a group of four, we should come back to this action, we're gonna have a group of four and we're gonna meet probably every six to eight weeks, and that is one from ED, one from anesthetics, one's from orthopedics and one from general surgery. Um, a, to look at the cases that are highlighted, cos although we're currently doing it, it's not being done with all four specialties, so we're, that's starting next month. Uh, to highlight those again, to take to the multi-disciplinary meetings. (Consultant) |
|  | We are (not) intending to contribute more towards resources and staffing to support trauma care and the transition to a MTC. | 6 | Consultant (3), Manager, Nurse (2) | We do have a, uh, another proposal for a coordinated hospital trauma response, and it's good to go, and it's gonna have to happen because of, um, changes that are happening within the emergency department, it is going to have to happen, um, so the timing around major trauma center is, is good from that perspective. (Consultant)  And, you know, I have risk-assessed it and it has, it has um, gone, you know, to my manager and upwards, but because we are moving, there'd be no plans to put extra oxygen points in. (Nurse) |
|  | I’m intending to discuss the trauma service with my colleagues | 1 | Nurse | No, [I haven't discussed it] none of my immediate colleagues, but I will now. (Nurse) |

| **TDF Domain** | **Identified Theme** | **Frequency (*n* transcripts)** | **Interviewees (Roles, *n*)** | **Sample Quote** |
| --- | --- | --- | --- | --- |
| **Knowledge** | Others have variable or limited knowledge of trauma | 5 | Consultant (3), Manager (2) | but the level of knowledge that people have of major trauma, of a major trauma center, and the implications of being a major trauma center, uh, are, are, are limited just now, but that's, that's what, something we'd need to work on (Manager) |
|  | I do not know what the resource requirements are for current trauma care or for becoming a MTC | 4 | Consultant (2), Manager (2) | I'm quite sure we don't have the, the resources in terms of staffing and infrastructure, um, but I don't know what those are yet, because we haven't calculated that, but we're just in the process of doing that. (Manager) |
|  | I know about trauma care and how to manage trauma patients | 5 | Consultant (4), Registrar | I think the basics of the, the skills and knowledge, um, that are required for the management of major, major trauma patients, are very much established in, in what we do in critical care. (Consultant) |
|  | There are (no) credible guidelines or algorithms for trauma patients at this hospital which improve patient care | 10 | All | Uh, I think it's credible, yes, uh, yes. I understand it was based on the English quality framework, so, uh, yeah, I think it's reasonably credible. (Manager)  I think they're still work in progress. (Consultant) |
|  | I keep up to date with evidence for major trauma care | 9 | Consultant (4), Registrar (2), Manager (2), Nurse | ...certainly more so recently. Uh, because of the, the development. (Manager) |
|  | Knowledge about the challenges of trauma care is improving | 1 | Consultants | No, I think that the, the evolution over the last six, seven, eight years has been miraculous, the evolution over the last year has been phenomenal . Probably the biggest benefit that I've had is that they've now seen what it's like out there, so when I bring patients back in, um, they are so much more constructive, um, about, they're not going on about why a blood pressure wasn't done, or this wasn't done, or this was done, or whatever, they understand how difficult and dangerous and demanding it can be out there. (Consultant) |
|  | Knowledge of the working environment is important | 2 | Nurse, SpR | and if you're, you work in the ward and you know where the things are that you need it shouldn’t become an issue… (Nurse) |
|  | Information and data is important to my role in trauma care | 3 | SpR, Cons, Manager | the more information we can get about what we're getting in and times etcetera, and the patient's condition, the easier it is. (Registrar) |

| **TDF Domain** | **Identified Theme** | **Frequency (*n* transcripts)** | **Interviewees (Roles, *n*)** | **Sample Quote** |
| --- | --- | --- | --- | --- |
| **Memory, Attention and Decision processes** | There are numerous potential distracting priorities at the same time as trauma that do not allow me to do my job and impact on patient care | 9 | Consultant (3), Manager (2), Nurse (2), Registrar (2) | we're so busy elsewhere dealing with cases that shouldn't be coming through the emergency department in order to keep the department safe (Registrar) |
|  | Being able to manage uncertainty and make decisions is important to trauma care | 3 | Consultant, Registrar, Nurse | So yeah I, I am a, I think it's important that at a certain level you are able to deviate from guidelines if it's required and you can balance risks and benefits. (Consultant) |
|  | Decision making is affected by some services not being available 24/7 | 1 | Consultant | the control centers that they know, that they can utilise is whenever they need to, because we don't have the resources to run twenty-four seven for the trauma team, they'll sometimes forget about the trauma team ‘cos they're not there all the time. (Consultant) |
|  | Guidelines help decision making | 2 | Consultant, Registrar | I think certainly they, um, aid confidence in what you already think is the best management for the patient, I think they make, um, the pathway that the patient goes through much, um, more streamlined, I think there's less, um, rule, there's less options for, for, you know different opinions in the management of a patient, and I think it does standardise it, make it, ultimately makes the patient care better. (Registrar) |
|  | Separating the medical and trauma presenting as unscheduled care will allow appropriate teams to deal with them | 1 | Manager | I would say the way forward is about separating out the, what is really a sort of good old-fashioned general medical presentation, complex and troublesome for the patient nonetheless, but um, uh, fighting to keep a limb alive, or a person alive, uh, as a consequence of being cut out of a vehicle will naturally take precedence and therefore being able to separate these out allows the two, allows the appropriate teams to deal with them. (Manager) |

| **TDF Domain** | **Identified Theme** | **Frequency (*n* transcripts)** | **Interviewees (Roles, *n*)** | **Sample Quote** |
| --- | --- | --- | --- | --- |
| **Motivation and Goals** | We should aim to deliver our best care and improve on it | 6 | Consultant (2), Registrar (2), Manager, Nurse | I think providing a great service to our patients, I think, um, is something that we should all strive for. (Registrar) |
|  | Goals related to trauma care should be a high priority | 6 | Consultant (4), Registrar, Manager | Major trauma care takes priority. First and foremost. (Registrar) |
|  | I do (not) know about goals for developing trauma services | 10 | All | ...that's why we're putting together the, the development program. Uh, so that we will be ready, or as ready as we can be, uh, by the end of two thousand sixteen…. (Manager)  Uh, I'm not aware of goals. I'm aware that some work has been done on the framework, um, in terms of what's been laid down in the trauma document and as I understand that was pretty much lifted from the document south of the border. (Consultant)  ...then I guess having that, if we all know what the, what our timelines are...that becomes the end point...it then becomes just A to Z, and it within the timescale. (Manager) |
|  | Achieving goals depends on the motivation of those involved, which is positive/negative | 10 | All | It's gonna be hard work to move the agenda forward unless they all realise how important a trauma agenda is for the whole of [local health board] and the whole of [this part of Scotland], not only trauma patients, because otherwise we'll be a [district general hospital]. (Consultant) |
|  | Our service is affected positively/negatively by targets and goals imposed from government level | 7 | Consultant (3), Registrar, Manager (2), Nurse | Whereas the vast majority of targets which are used as a stick if you like, to beat, a, uh, NHS board with, or indeed to allocate reward, are based upon elective waiting lists, rather than outcomes, and specifically outcomes of unscheduled care, which I think are, uh, very much the poor cousin. (Consultant) |
|  | Departments and individuals have a high/low motivation for trauma care | 8 | Consultant (4), Registrar (2), Manager (2) | Yeah, timeliness, attendance...essentially it was because the leadership of surgery didn't buy into this as a concept. And that's still the position. (Consultant)  Outwith the hospital it's really variable actually, outwith the emergency department it's really variable. The attitude that we get when we pre-alert folk about major trauma. Um, and it depends on who's on-call often as well (Registrar) |
|  | I am motivated to be involved in the transition to MTC | 10 | All | I very much feel that we should become a major trauma center, I feel I am committed to doing whatever I could do to facilitate that process, and I would hope that that view is shared by other people. (Consultant) |

| **TDF Domain** | **Identified Theme** | **Frequency (*n* transcripts)** | **Interviewees (Roles, *n*)** | **Sample Quote** |
| --- | --- | --- | --- | --- |
| **Optimism** | I’m optimistic/pessimistic about the changes being made and the role of major trauma at the hospital | 10 | All | I, I'm highly confident that we can. I'm highly confident that we could do it. (Manager)  This worries me about how we're gonna become a major trauma center. (Registrar) |
|  | My optimism/pessimism is conditional upon availability of necessary resources | 4 | Consultant, Manager (2), Registrar | ...part of that will be to, um, develop the skills of the clinician and recruit the, to recruit, um, people who can actually provide the service. Because I'm not confident that we have enough people who are able to deliver that service. And that's one of the major challenges that we have. (Manager)  ...if we had everything that I've just described to you, plus the authority to make it happen, I reckon we could probably have it up and running by this time next year. (Consultant) |

| **TDF Domain** | **Identified Theme** | **Frequency (*n* transcripts)** | **Interviewees (Roles, *n*)** | **Sample Quote** |
| --- | --- | --- | --- | --- |
| **Reinforcement** | I am (not) aware of any material rewards for becoming a trauma center. | 10 | All | Well, we're advized by the government that there isn't additional resources. (Manager)  Hopefully if we were a major trauma center we'd get a bit more funding as well to, to expand our roles. (Registrar) |
|  | Elective work is rewarded more than emergency work. | 1 | Cons | It does in that, um, there has been for some considerable period of time, preferential funding allocated to, uh, deal with these waiting list targets and suchlike, so targets around, uh, elective work, rather than, um, resourcing, uh, areas which deal more with unscheduled work. (Consultant) |
|  | There is no formal reward for activities such as training. | 1 | Nurse | Well, you don't get a certificate or anything at the end of it, but yeah, it's, [training is] done in the unit. Yeah. (Nurse) |

| **TDF Domain** | **Identified Theme** | **Frequency (*n* transcripts)** | **Interviewees (Roles, *n*)** | **Sample Quote** |
| --- | --- | --- | --- | --- |
| **Skills** | In general, there are (not) sufficient levels of the necessary technical skills at this hospital to provide major trauma care | 10 | All | I mean certainly I think experience is lacking, it is just one, uh, is one aspect, um, I think certain courses I think would be useful to do, but I think the fact that, um, I don't see a huge number of trauma cases but I think my experience is much less of that than someone who is based at a trauma center at the moment. (Registrar)  With a long inning in neurosurgery almost spanning three decades, I feel able to deal with most trauma situation. I also had an additional (inaudible) of a spinal orthopedic, uh, fellowship. And therefore I feel able to deal with most of the spinal trauma also. (Consultant) |
|  | There are (not) sufficient amounts of teaching and training in trauma care at [this hospital] | 8 | Consultant (2), Manager (2), Registrar (2), Nurse (2) | it would be much nicer if we, if we prioritized and nurses did have proper training. Things like the trauma, rather than how to clean a bed frame properly. (Nurse)  I haven't been involved in it myself cos I've been working (Registrar)  Um, from, we do simulation training now in [this hospital], it's a new thing a couple of the consultants are keen to do. (Registrar) |
|  | Maintaining skills is important as well as developing them | 4 | Consultant (2), Registrar, Manager | Um, trauma for those, because it doesn't happen, a number of times every day, there is an issue of maintaining skill and making sure that folk are adequately prepared to be able to mount the right response when it is required. (Manager) |
|  | We can improve our care by learning skills from others both within and outwith trauma. | 7 | Consultant (3), Registrar, Manager (2), Nurse | Some surgical procedures and, I think that the, it would be of benefit for us to see how it is done elsewhere. (Consultant)  Could be a good way, but, well, either the individuals working or leading this change may go and work in another place and learn from others what they had problems, how they solve it, or people from other established centers coming and assisting us, supporting us in the initial phase of this transition. Both ways it could be worked at, but I think we won't need to reinvent the wheel as they say in this, uh, we can learn from others' successes and failures, rather than having to do it ourselves, everything. (Consultant) |
|  | Skills in major trauma care would be better if this hospital were to become an established MTC. | 7 | Consultant (2), Registrar (2), Manager (2), Nurse | I think that for trainees, I think it would be hugely useful, I think the experience they would gain from it, I think the decision-making skills, I think the technical skills, um, I think um, I think that that would be fantastic… (Registrar) |
|  | Managing trauma patients is routine | 7 | Consultant (3), Registrar, Manager, Nurse | Yeah. Yeah. I mean, well obviously all the staff are trained up on spinal injuries and, and major injuries like that. They are routine. (Nurse) |
|  | There are sufficient levels of the necessary non-technical skills at this hospital to provide major trauma care | 8 | Consultant (4), Registrar, Manager, Nurse (2) | I think I'm a good communicator, I get on really well with the staff here. (Registrar) |
|  | If you have experience and skills there is less need for protocols and guidelines –but these help if you don’t | 2 | Cons, SpR | I think certainly when more junior members of the team are involved I certainly think a guideline at the very least would be very helpful in allowing consistent practice, and at the best level. (Consultant) |
|  | It is important not to lose skills from elsewhere by focusing on trauma | 1 | Manager | Themes of things that I can think of would be around how do we disaggregate this then from our existing services, without detriment to other services because we almost co-relocate a, a bit of the service from somewhere else, so if we dislocated it and moved it to develop into something else, what does that mean? Do we lose skill that we need in other bits of our service, as a consequence of doing that?  (Manager) |
|  | Teaching and training improves performance | 2 | Cons, Nurse | So hopefully if there was input into, to more staffing and more training then patients would benefit from that, in their care. Their outcomes. (Nurse) |
|  | It is important to train with the people you work with | 1 | Cons | ...we've all trained together, worked together… (Consultant) |
|  | My skillset is used in stressful and emotive situations | 1 | Cons | ...resuscitating two-year olds or three-day olds, um, and sixteen-year olds who hung themselves, etcetera, etcetera, and as I said I do play, these things do play on my mind, I, I probably if I got analysed would be put down as post-traumatic stress cos I do three hours sleep a night or something... (Consultant) |
|  | Trialling or practising aspects of becoming a MTC would be helpful | 4 | 2 Nurse, Cons, Manager | [trialling the MTC] It will be helpful. I don't think it is a must, but I think it is ideal, and possibly help identify the things we need to put in place before going full-blown. (Consultant) |

| **TDF Domain** | **Identified Theme** | **Frequency (*n* transcripts)** | **Interviewees (Roles, *n*)** | **Sample Quote** |
| --- | --- | --- | --- | --- |
| **Social Influences** | Management, nursing and medical staff do not work well together at present | 3 | Consultant, Nurse, Registrar | a lot of the issues that surround us at the front lines, seem to be belittled or ignored by senior management. (Consultant) |
|  | There is variation amongst the views of myself and my colleagues about the transition to a MTC. | 9 | Consultant (4), Manager (2), Nurse (2), Registrar | I don't think a lot of people understand the implications of not becoming a major trauma center, for [local health board]. And if they did, I think a lot more people would, would put their hat in the ring. (Consultant)  [how committed are your colleagues to becoming a MTC?] The same. But they share my reservations, so, you know, there's heaps of reservations along the way, but absolutely committed. Just wish we saw that level of commitment from, from everyone. (Consultant) |
|  | Authority and support from leadership figures is important in the current and future care of trauma patients | 8 | Consultant (4), Registrar (2), Manager (2) | At a cost of repetition, [anesthesiologist], [Emergency Room consultant], and [trauma surgeon] I believe are the leaders who are driving this forward. And we will be swinging on their tail, as they say. (Consultant) |
|  | Knowing your colleagues well and understanding their strengths and limitations in an established team improves patient care. | 7 | Consultant (3), Registrar (2), Manager (2) | [this city] is still fairly small, very large village, where everyone knows everyone, they all know me when I arrive on the scene… (Consultant) |
|  | We need to work together with national and regional health bodies and outside organizations when planning the transition to MTC. | 4 | Consultant (2), Manager (2) | Cos we work with oil and gas. I mean if we can't work with oil and gas in [this part] of Scotland… (Consultant)  it will allow us to develop really, it provides a stimulus for us to develop good relationships with our, [local health boards], uh, to, uh, attract the activity from the [region of Scotland]. (Manager) |
|  | My practice is (not) influenced by guidelines and protocols | 8 | Consultant (3), Registrar (2), Manager, Nurse (2) | I believe in the evidence-based medicine and therefore these are very valuable tool as far as, what I cannot substitute is that human intellect still has overriding importance, to use this judiciously, but they are definitely a good skeleton to work with. (Consultant)  I don't know if they [guidelines] would make a difference or not, but if there was evidence that it would then I'd be all for it. (Registrar) |
|  | Good teamwork is important to the current and future care of trauma patients | 10 | All | ...we work as a unit across the floor and help each other out… (Nurse) |
|  | I practice in the same way as my colleagues and peers | 4 | Consultant (2), Registrar, Nurse | Yeah, yes I think, I think they do [provide major trauma care in the same way].  (Nurse) |

| **TDF Domain** | **Identified Theme** | **Frequency (*n* transcripts)** | **Interviewees (Roles, *n*)** | **Sample Quote** |
| --- | --- | --- | --- | --- |
| **Social and Professional Role** | I should play a role in the initial assessment and resuscitation of the patient | 6 | Consultant (4), Registrar (2) | I think [surgeons] should be involved from the outset when they arrive in hospital, um, and I think they should be involved in the decision making for that patient. (Registrar) |
|  | Someone should lead and coordinate the care of trauma patients through hospital | 5 | Consultant (2), Registrar (2), Nurse | I think that that would ideally work best because the ownership for the care of that patient would be coordinated by one member or one team, um, which would make it I think much easier to manage them (Registrar) |
|  | I should play a role in the transition to major trauma center | 8 | Consultant (4), Registrar, Manager (2), Nurse | I now see it as quite a large part of my role, the, uh, because the development of [this hospital] as a major trauma center is regarded as a high priority by the board. (Manager) |
|  | Management and politicians play a positive/negative role in steering the trauma service. | 8 | Consultant (4), Registrar, Manager, Nurse (2) | I think, um, from management levels, I'm not sure how, I get a feeling there's reluctance but I don't know if that is, is true or not. (Registrar)  Well, I mean it's encouraged by, um, by the government, because it's government policy. Uh, so that is a large, uh, encouragement. Um. It is now encouraged by, uh, the executive team, and, and our board. (Manager) |
|  | I do (not) see trauma as a large part of my role | 10 | All | A huge part of my role, it's exactly why I chose to do emergency medicine, it's exactly what interests me, um, and without the possibility of seeing major trauma I probably wouldn't choose to do emergency medicine. (Registrar)  I think that, uh, in my role, uh, trauma, as in emergency department medicine is actually only one bit of everything that I do, so, I also have intensive care, theatres, and about eight other services that are all reporting, uh, within my division. (Manager) |
|  | I do not fully appreciate the extent of others’ roles | 3 | Cons, SpR, nurse | As a trainee I might not be proviso to that… (Registrar) |
|  | I play a role in local and national boards/organizations | 1 | Cons | ...having worked with the Department for Health and the Department for Transport and now Ministry of Defence things like that… (Consultant) |
|  | I currently play a role in improving the service the hospital provides to trauma patients | 3 | Cons, manager, nurse | Yes, well we, I, um, [trauma surgeon] organizes them, so basically yeah, um, I go to nearly all of them, um, I, I'm the local coordinator for the STAG data, so I go through the STAG data with our STAG nurse, audit nurse, um, but again, finding a multi-specialty environment is, is the key issue there (Consultant) |
